# Metagenomic Sequencing with Strain-Level Resolution Implicates Uropathogenic *E. coli* in Necrotizing Enterocolitis and Mortality in Preterm Infants

**DOI:** 10.1016/j.celrep.2016.03.015

**Published:** 2016-03-17

**Authors:** Doyle V. Ward, Matthias Scholz, Moreno Zolfo, Diana H. Taft, Kurt R. Schibler, Adrian Tett, Nicola Segata, Ardythe L. Morrow

**Affiliations:** 1 Center for Microbiome Research, University of Massachusetts Medical School, Worcester, MA 01655, USA; 2 Centre for Integrative Biology, University of Trento, Trento, TN 38123, Italy; 3 Department of Pediatrics, Cincinnati Children’s Hospital Medical Center, Cincinnati, OH 45229, USA; 4 Co-first author; 5 Co-senior author

## Abstract

Necrotizing enterocolitis (NEC) afflicts approximately 10% of extremely preterm infants with high fatality. Inappropriate bacterial colonization with Enterobacteriaceae is implicated, but no specific pathogen has been identified. We identify uropathogenic *E. coli* (UPEC) colonization as a significant risk factor for the development of NEC and subsequent mortality. We describe a large-scale deep shotgun metagenomic sequence analysis of the early intestinal microbiome of 144 preterm and 22 term infants. Using a pan-genomic approach to functionally subtype the *E. coli*, we identify genes associated with NEC and mortality that indicate colonization by UPEC. Metagenomic multilocus sequence typing analysis further defined NEC-associated strains as sequence types often associated with urinary tract infections, including ST69, ST73, ST95, ST127, ST131, and ST144. Although other factors associated with prematurity may also contribute, this report suggests a link between UPEC and NEC and indicates that further attention to these sequence types as potential causal agents is needed.

## Graphical abstract

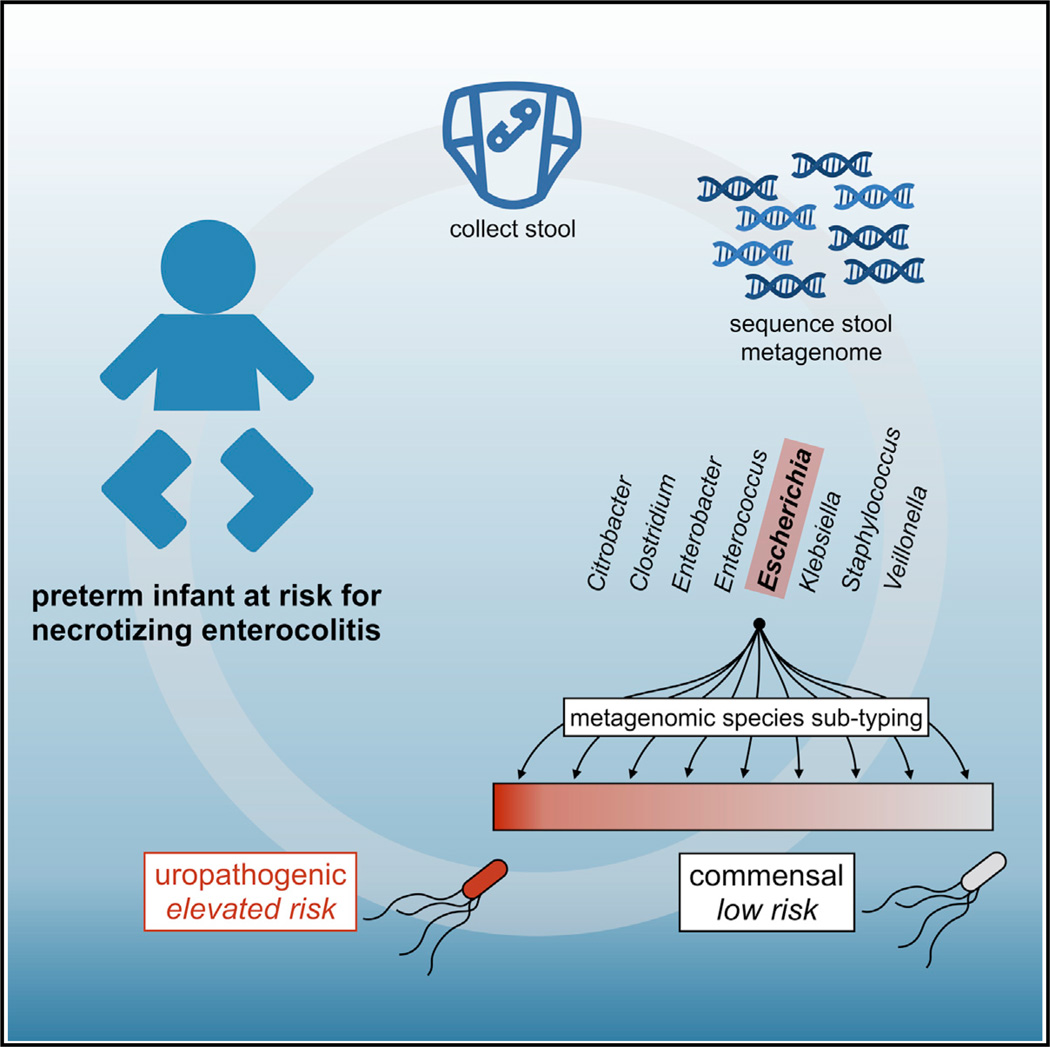

## INTRODUCTION

Necrotizing enterocolitis (NEC) is a leading cause of morbidity and mortality in preterm infants. One in fourteen infants born prior to 32 weeks of gestation develop NEC, and nearly one-third of cases is fatal (Neu and Walker, 2011). The etiology of NEC is not confidently established but appears to result from a hyperinflammatory response to the gut microbiota (Nanthakumar et al., 2011; Neu and Walker, 2011). For years, research on NEC focused on identifying pathogens responsible for triggering NEC onset (Boccia et al., 2001; Hoy et al., 2000). With the advent of high-throughput sequencing, the focus shifted toward identifying microbial community dysbiosis that predisposes infants to develop NEC (Claud et al., 2013; La Rosa et al., 2014; Mai et al., 2011; Morrow et al., 2013; Torrazza and Neu, 2013; Wang et al., 2009; Zhou et al., 2015).

Relative to term infants, the enterocytes of preterm infants exhibit excessive toll-like receptor 4 (TLR4) signaling in response to lipopolysaccharide (LPS)-bearing organisms (Nanthakumar et al., 2011). The most abundant LPS-bearing bacteria in the pre-term infant are of the phylum Proteobacteria, family Enterobacteriaceae. Some have reported that Proteobacteria are more abundant at the time of NEC onset (Wang et al., 2009), whereas others have reported a surge in Proteobacteria a week or more prior to NEC onset (Claud et al., 2013; Morrow et al., 2013; Torrazza and Neu, 2013; Zhou et al., 2015). These reports support the view that an excessive quantity or increase in Proteobacteria triggers a hyperinflammatory response that leads to NEC. However, many preterm infants are highly colonized by Proteobacteria and experience a surge in this phylum without developing NEC (La Rosa et al., 2014). Thus, a generalized Proteobacteria dysbiosis alone does not adequately explain this disease.

NEC often occurs in outbreaks (Boccia et al., 2001; Mein-zen-Derr et al., 2009), and investigations using culture-based techniques have reported that Enterobacteriaceae members *Escherichia coli*, *Klebsiella pneumoniae*, and *Enterobacter cloacae* were most often associated with NEC (Boccia et al., 2001; Hoy et al., 2000). The most recent investigations of NEC have used culture-independent 16S rDNA community profiling (Claud et al., 2013; La Rosa et al., 2014; Mai et al., 2011; Morrow et al., 2013; Torrazza and Neu, 2013; Wang et al., 2009 Zhou et al., 2015), which lacks the ability to resolve strains with “pathogenic potential” from non-pathogenic members of a lineage. A recent shotgun metagenomic study with five temporally clustered cases of NEC and five healthy infants provided strain-level resolution but did not find a specific, “genetically distinct” pathogenic strain associated with the case cluster (Raveh-Sadka et al., 2015), and cultivation of Enterobacteriaceae strains associated with 15 episodes of suspected NEC (eight confirmed) reached a similar conclusion (Hoy et al., 2000).

We analyzed deep shotgun metagenomic sequencing of fecal samples collected from 144 preterm infants in 3 neonatal intensive care units and 22 term infants. We applied an assembly-free, pangenome-based computational analysis to determine the *E. coli*-specific gene content of strains within the infant microbiomes. Accessory genes involved in iron acquisition, phosphotransferase systems (PTSs), and D-serine metabolism were characteristic of strains associated with NEC risk. Additional analysis characterized the risk strains as uropathogenic *E. coli* (UPEC) (Flores-Mireles et al., 2015; Wiles et al., 2008; Zhang and Foxman, 2003). We present evidence that (i) colonization by UPEC is a risk factor for development of NEC and (ii) UPEC correlated with death as an outcome. These findings suggest an association between UPEC and NEC and provide a foundation for advancing the epidemiology of NEC.

## RESULTS

### Subjects and Overview

We generated metagenomic shotgun sequence data from 144 preterm infants less than 30 weeks gestational age (GA) and 22 term infants greater than 37 weeks GA with stool samples collected between days 3 to 22 of life (Tables 1 and Table S1; Figures S1 and S2). We divided the collection period into three windows (days 3–9, 10–16, and 17–22) and selected the latest postnatal sample per infant per window for inclusion in community analysis. NEC cases and controls had a similar average day of life of sample collection within each of the three windows analyzed. We investigated the prevalent Enterobacteriaceae species in relation to risk of NEC. These results led us to functionally subtype *E. coli* and associate UPEC with NEC and infant death.

### Infant Microbiome at Days 3–9 Postpartum

**Figure 1:**
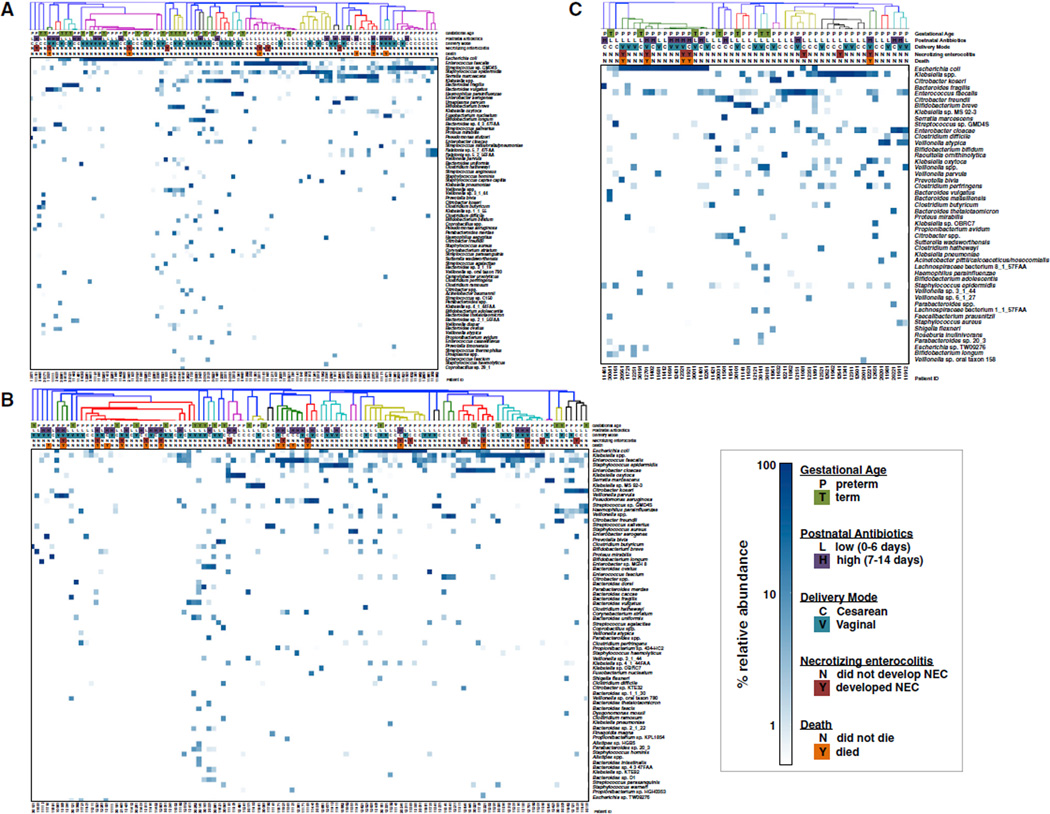
Species Composition and Relative Abundance in the Infant Gut (A) Days 3–9 postpartum. (B) Days 10–16 postpartum. (C) Days 17–22 postparum. Species are ranked top to bottom by average relative abundance across samples. Species presented exhibit a minimum average relative abundance of 0.2% or achieve at least 5% relative abundance in at least one sample (complete abundance data in Table S1). Samples were hierarchically clustered with Bray-Curtis dissimilarity. Color indicates relative abundance on a logarithmic scale.

**Figure 2:**
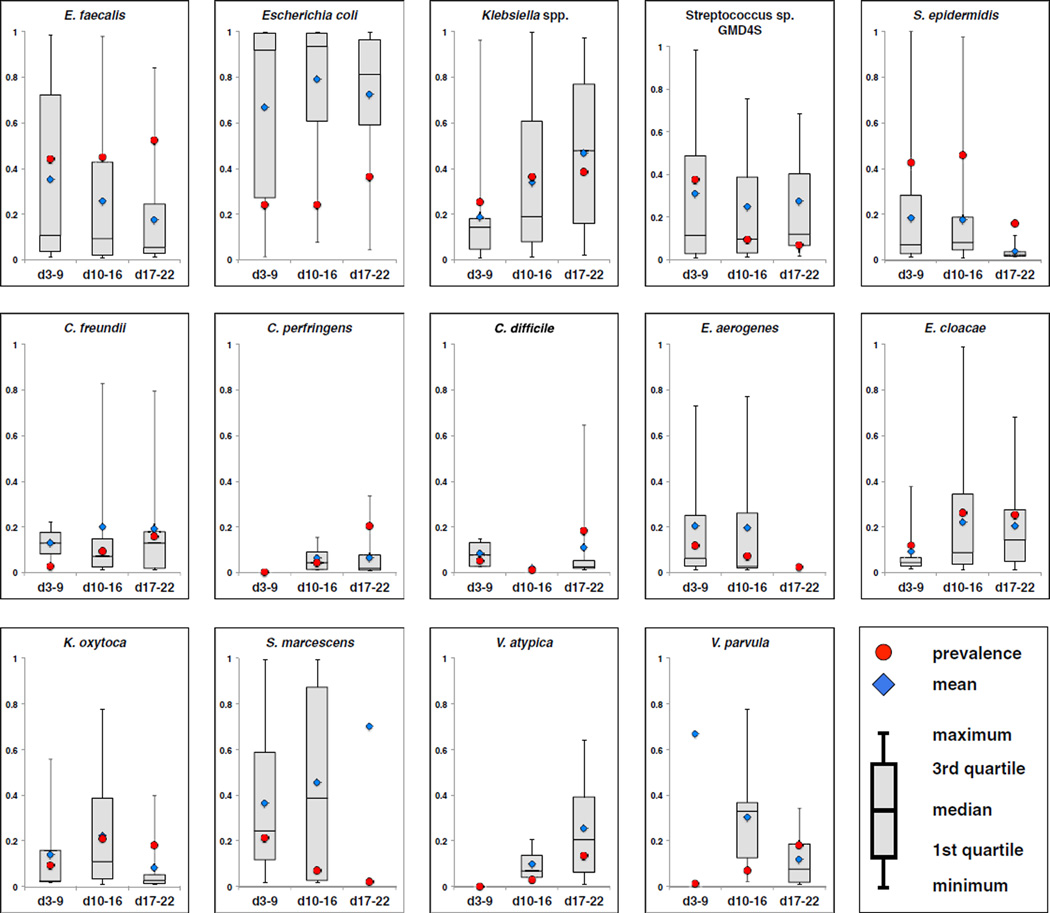
Relative Abundance and Prevalence of the Most Frequently Occurring Species in the Early Preterm Infant Microbiome Fourteen of the most prevalent species observed in the preterm infant microbiome are presented. *E.coli* is an outlier with a median relative abundance exceeding 0.80 in each of the three collection windows presented. Relative abundance calculations include only samples where the infant carried the organism with at least 1% relative abundance. Prevalence is indicated as red circle, mean relative abundance as a blue diamond, and median relative abundance as box and whisker plot.

Samples from 97 infants, 75 preterm and 22 term, were collected days 3–9 postpartum (median day of sample collection = 7). 8 of the preterm infants later developed NEC. MetaPhlAn (v2.0) (Segata et al., 2012; Truong et al., 2015) was applied in order to determine the relative abundance of species present (Figure 1A and Table S1). Defining carriage to be when a sample contains a species present at a minimum of 1% relative abundance, we determined the most commonly carried species in this sampling window. In descending order, *Enterococcus faecalis* occurred in 33 of 75 preterm infants (a prevalence of 44%) followed by *Staphylococcus epidermidis* (43%), *Streptococcus* sp. GMD4S (37%), *Klebsiella* spp. (25%), *E. coli* (24%), *Serratia marcescens* (21%), *Enterobacter aerogenes* (12%), and *Enterobacter cloacae* (12%) (Figure 2, red circles).

In many samples, a single species dominated the community. We calculated the median relative abundance (MRA, scaled in the [0.0–1.0] interval) of the most commonly carried species, excluding samples in which the species were not carried. The most abundant species, ordered by MRA, were *E. coli* (0.92), *S. marcescens* (0.24), *Klebsiella* spp. (0.14), *Streptococcus* sp. GMD4S (0.12), and *E. faecalis* (0.11) (Figure 2).

Term infant samples were more diverse than preterm infant samples, as assessed with Shannon’s index (SI) (Figure S3A; SI = 1.59 ± 0.95 and 1.1 ± 0.77, respectively; p = 0.015). To identify taxa that were significantly enriched in either term or preterm infants, we used LEfSe (Segata et al., 2011) (Figures S4A–S4C). Taxa enriched in term infant samples included the phyla Acti-nobacteria (e.g., *Bifidobacterium* species) and Bacteroidetes, as well as some of the Firmicutes, including the classes Clostridia, Erysipelotrichia, and Negativicutes (e.g., *Veillonella*). In contrast, taxa enriched in preterm infant samples included Firmicutes classes Bacilli (e.g., *S. epidermidis*) and Lactobacillales (e.g., *E. faecalis*) as well as the Gammaproteobacteria genus *Enterobacter*.

In preterm infants, early empirical antibiotic prophylaxis consisted of ampicillin and gentamicin. The number of days of antibiotic treatment during the first 14 days of life was used to stratify infants. We observed few, if any, significant differences in microbiome composition between preterm infants with no postnatal antibiotic treatment and infants with only a few days of treatment (data not shown). Thus, we compared preterm infants who received 0–6 days treatment (low) to those who received 7–14 days treatment (high) during the first 2 weeks of life (Figures 1A, S3, and S4D–S4E). Diversity did not differ significantly comparing low and high antibiotic treatment (SI = 1.06 ± 0.79 and 1.18 ± 0.7, respectively; p = 0.582). Infants who received low treatment were enriched in the class Bacilli; high treatment infants were enriched in the genus Bacteroides (Figures S4D and S4E). These differences may be due to antibiotic treatment, or, alternatively, infants with these profiles may differ in their likelihood of treatment with antibiotics.

There were 46 Cesarean births and 29 vaginal births in the pre-term infants with days 3–9 samples. Diversity did not differ significantly by delivery mode (SI = 1.21 ± 0.81 versus 1.02 ± 0.73; p = 0.305). However, Bacilli were enriched in Cesarean births (Figures S4F and S4G).Preterm infants who developed NEC had similar diversity in comparison to preterm controls (SI = 1.11 ± 0.79 versus 0.96± 0.56; p = 0.597), and there were no notable differentially enriched taxa between these groups (data not shown).

### Infant Microbiome at Days 10–16 Postpartum

Infant samples were collected during days 10–16 from 96 preterm and 18 term infants (median day of sample collection = 13); 15 preterm infants later developed NEC. (Figure 1B, Table S1). The prevalence of the most commonly carried organisms was *S. epidermidis* (46%), *E. faecalis* (45%), *Klebsiella* spp. (36%), *E. cloacae* (26%), *E. coli* (24%), and *K. oxytoca* (21%) (Figure 2). However, the species with the highest MRA were *E. coli* (0.93), *S. marcescens* (0.39), *V. parvula* (0.33), *Klebsiella* spp. (0.19), *K. oxytoca* (0.11), and *Streptococcus* sp.GMD4S (0.12) (Figure 2).

Again, term infant samples were more diverse than preterm infant samples (SI =1.82 ± 01.1 versus 1.13 ±0.76; p = 0.002). Pre-term infants delivered vaginally tended to have lower diversity than Cesarean births (SI = 0.96 ± 0.68 versus 1.25 ± 0.79; p = 0.061), and infants with high antibiotic treatment tended to have lower diversity than those with low antibiotic exposure (SI = 0.92 ± 0.81 versus 1.22 ± 0.71; p = 0.073). However, infants who developed NEC did not significantly differ in diversity from preterm controls (SI = 1.01 ± 0.92 versus 1.15 ± 0.72; p = 0.524).

Term infants were enriched in nearly all taxa, except that the species *S. epidermidis*, *E. faecalis*, *E. cloacum*, and *S. marcescens* were all enriched in preterm infants in this second collection window (Figure S5A and S5B). Differences in delivery mode or antibiotic treatment indicated few differences in these samples (Figure S5C and S5D). *Streptococcus* and the order Bacteroidales were enriched in infants with high antibiotic exposure (Figure S5C and S5D). *Citrobacter* and *Klebsiella* were enriched in Cesarean delivered infants (Figure S5E and S5F). NEC infants did not exhibit notable, differentially enriched taxa relative to preterm controls (data not shown).

### Infant Microbiome at Days 17–22 Postpartum

44 preterm infants and 7 term infants were included in the days 17–22 collection window (median day of sample collection = 20); 7 preterm infants later developed NEC (Figure 1C; Table S1). The most prevalent organisms were *E. faecalis* (52%), *Klebsiella* spp. (39%), *E. coli* (36%), *E. cloacae* (25%), *C. perfringens* (20%), *C. difficile* (18%), *K. oxytoca* (18%), *V. parvula* (18%), *C. freundii* (16%), *S. epidermidis* (16%), and *V. atypica* (14%). Again, the species with the highest MRA provided a different picture—*E*. *coli* (0.81), *Klebsiella* spp, (0.48), *V. atypica* (0.21), *E. cloacae* (0.14), *C. freundii* (0.13), and *Streptococcus* sp. GMD4S (0.12) (Figure 2).

Term infant samples were again more diverse than the preterm infant samples (SI = 2.35 ± 1.2 versus 1.24 ± 0.69; p = 0.0017). Similar to the days 10–16 samples, diversity did not differ by delivery mode (SI = 1.11 ± 0.67 versus 1.35 ± 0.67; p = 0.253). However, infants with high antibiotic treatment were markedly less diverse (SI = 0.91 ± 0.67 versus 1.39 ± 0.64; p = 0.032). Samples from infants who developed NEC tended to be less diverse than preterm controls, but the difference was not significant (SI = 0.87 ± 0.63 versus 1.32 ± 0.68; p = 0.122).

Term infants were enriched in nearly all taxa except *E. faecalis* and *Streptococcus*, which were enriched in preterm infants (Figure S6A and S6B). Delivery mode indicated few differences, although *E. aerogenes* was enriched in Cesarean births (Figure S6C). Infants with high antibiotic treatment were specifically enriched in *E. coli* relative to low treatment infants who were enriched in the order Clostridiales, genus *Veillonella*, and *Klebsiella* spp. (Figure S6D and S6E). Finally, infants who developed NEC had less *Veillonella* and were specifically enriched in *E. coli* (Figure S6F and S6G).

### *E. coli* and NEC-Associated Death

Of 27 infants who developed NEC, 13 had *E. coli* at greater than 1% relative abundance in at least one sample collected from days 3–22 of life; 10 of the 13 infants died. The next most abundant species, carried in 12 infants (4 deaths), was *Klebsiella* spp.; 2 NEC cases carried both *E. coli* and *Klebsiella* spp. In infants who developed NEC, *Klebsiella* spp. carriage in at least one sample correlated with NEC survival (p = 0.057), whereas *E. coli* carriage correlated with death (p = 0.054).

### Defining *E. coli* Sub-types as a Risk Factor for NEC

**Figure 3:**
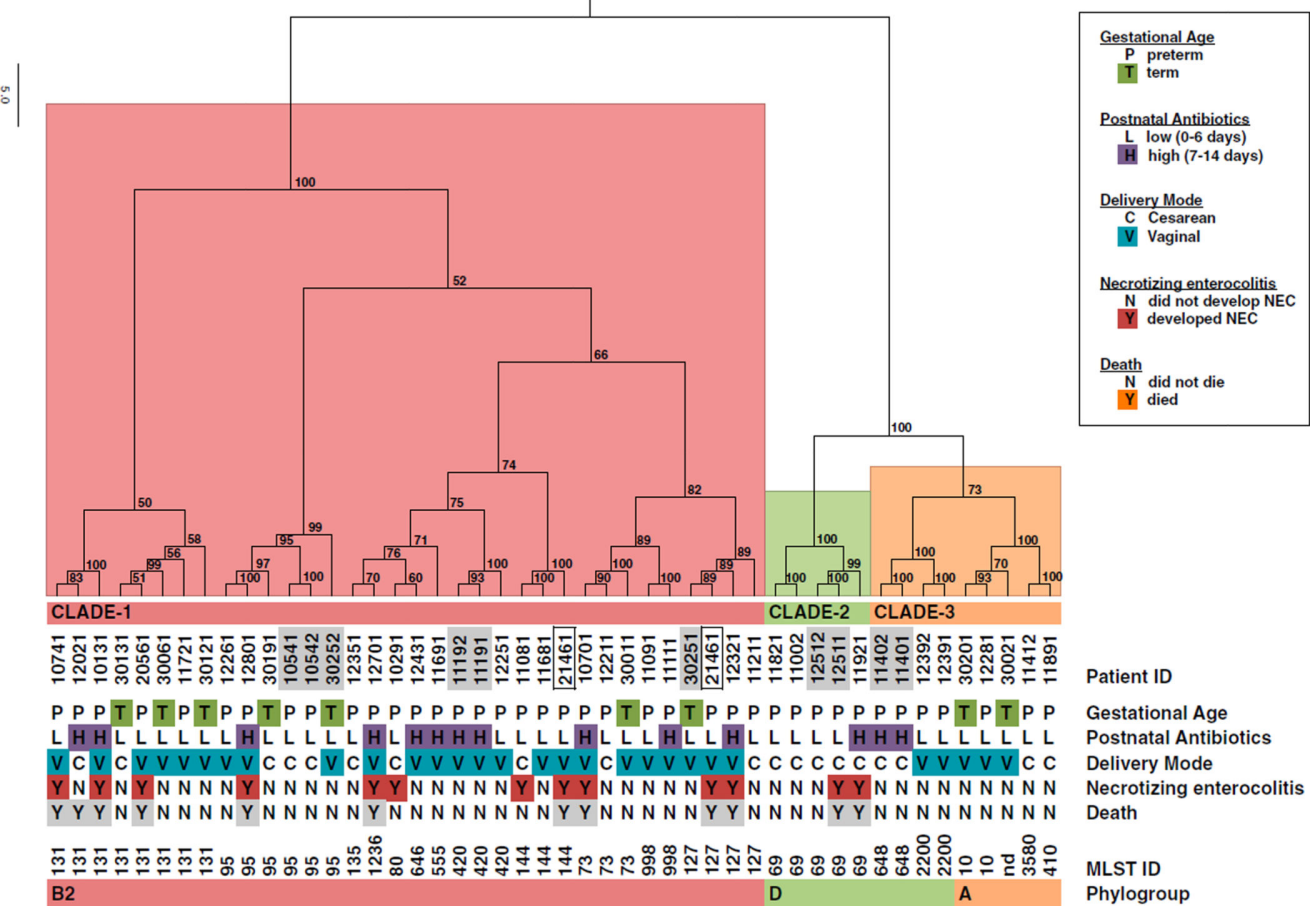
Functional and Metagenomic MLST Sub-typing of *E. coli* Strains Present in Metagenomic Samples Indicates UPEC Lineages Are Associated with NEC Risk *E. coli* accessory gene-content information (Table S2) was used to hierarchically cluster infant strains and indicated three distinct functional clades. Approximately Unbiased (AU) p value, a multiscale bootstrap resampling method (Shimodaira, 2004), indicates the strength of support for clusters. Clades 1 (red) and 2 (green) contain NEC cases. Metagenomically identified MLST IDs are indicated, and the corresponding phylogroup inferred from literature and available resources (http://mlst.warwick.ac.uk/mlst/). Consecutively numbered and shaded infant IDs indicate fraternal twin pairs. Boxed infant IDs indicate two strains of *E.coli* identified in temporally separated samples from a single infant (patient ID 21461).

Considering the prior association of Enterobacteriaceae with NEC, and our observations that *E. coli* was associated with NEC and NEC-associated death, we hypothesized that strain level differences among colonizing *E. coli* may further stratify NEC risk. To test this hypothesis, we developed assembly-free, metagenomic approaches to subtype *E. coli* by gene content (Scholz et al., 2016) and by multilocus sequence type (MLST) assignment (Figure 3, Tables 2, 3, S1, and S2). Both approaches indicated that the dominant *E. coli* strain-type in any single infant was constant over the collection period. Infant 21461 was an exception, providing two samples with each presenting a different dominant strain type. For association analysis, we considered genes that could be assigned to a KEGG Orthology (KO) group (Table S2) (Kanehisa et al., 2014). The UniProt Knowledgebase (http://www.uniprot.org/uniprot/) was used to infer *E. coli*-specific gene assignments from KO assignments. 919 of 3,004 total genes with KO assignments were considered accessory genes in that they were not found in all infant samples (Table S2). Strain-specific gene presence and absence profiles were used to hierarchically cluster the samples (Figure 3). Infants who developed NEC were observed throughout the large cluster (clade 1) and in a second smaller cluster (clade 2).

### *E. coli* Accessory Genes Associated with NEC-Containing Clades

In total, 120 genes significantly associated with clade 1 and clade 2,and 152 genes associated with clade 3 (p < 0.02) (Tables 2, 3, and S2). Because strains from infants that developed NEC tightly co-clustered with strains from infants who did not develop NEC (Figure 3)—and, thus, would have very similar gene content—we did not expect, and did not find, any *E. coli* genes significantly associated with development of NEC (data not shown). Genes involved in phenylalanine metabolism and the degradation of aromatic compounds (Díaz et al., 2001) were associated with clade 3. Phylogroup A strains exhibit capacity for aromatic acid utilization, whereas clinical isolates generally lack these capacities (Burlingame and Chapman, 1983). Functional gene categories significantly associated with clades 1 and 2 included iron acquisition and metabolism, D-serine detoxification and metabolism, and sugar-specific PTSs. These gene categories are found to be enriched in UPEC strains, where they most likely represent adaptation to the environment of the urinary tract (Brzuszkiewicz et al., 2006; Chen et al., 2006; Lloyd et al., 2007). Thus, we speculated that clade 1 and 2 *E. coli* strains may be functionally related to UPEC. The observed clade structure (Figure 3) also indicated *E. coli* subtypes within the three clades we defined. We sought to further resolve these subtypes so as to better define the *E. coli* lineages present in our cohort.

### Metagenomic MLST Analysis Indicates that Clade 1 and 2 Strains Are UPEC

We were able to assign an existing an multilocus sequence type (MLST) (Maiden et al., 1998) to most samples with at least 1% relative abundance of *E. coli* (Figure 3; Table S1). The assigned MLSTs are remarkable in their concordance with the clade structure obtained independently from accessory gene content—all MLSTs mapped to discreet subclades within our functionally defined clusters. Phylogroup B2 MLSTs 73, 95, 127, 131, 144, and 998 fall in clade 1. Clade 2 represents MLST 69 and is a phylogroup D type. Clade 3 contains representatives that have been recognized in animal hosts—MLSTs 648 and 2200 have been placed in phylogroup D (Ciccozzi et al., 2013; Giufreè et al., 2012; Wieler et al., 2011)—as well as phylogroup A strains.

The dataset contains five fraternal twin pairs (Figure 3, consecutively numbered, shaded infant IDs). The four preterm pairs each share the same MLST assignment, whereas the term pair differs. Preterm infant 12511 developed NEC and died, whereas the sibling twin, infant 12512, survived free from NEC.

We identified one potential strain turnover event in a single infant. *E. coli* from infant 21461 typed as ST127 in a sample from day 7 and as ST144 from a day 10 sample. Infant 21461 developed NEC and died on day 33. Both samples indicated greater than 95% relative abundance of *E. coli*. Each sample clustered by functional composition with other samples of the same MLST. In the association analyses presented, we have included the ST127 sample.

### UPEC as a NEC Pathogen and Risk Factor for Death

We identified UPEC MLSTs belonging to phylogroups B2 and D as associated with NEC risk. We then calculated the ORs regarding UPEC as a risk factor for NEC, NEC-associated deaths, and all deaths. In our primary analysis, we included 143 of the available 144 preterm infants less than 30 weeks GA (Table 4; Figure S2). The data from one preterm infant who survived free from NEC or death (12611) failed in *E. coli* analysis, allowing no conclusions about UPEC status for this infant to be made; thus, this infant was excluded from analysis. We defined controls as preterm infants who survived hospitalization free of NEC. The analysis included 27 NEC cases and 21 deaths (of which 15 deaths were NEC-associated and 6 were independent of NEC). UPEC was significantly associated with NEC, NEC-associated deaths, all deaths, and NEC or death, with unadjusted ORs of 4.1 (p = 0.003), 10.3 (p < 0.001), 5.7 (p < 0.001), and 3.4 (p < 0.007), respectively.

We then modeled NEC and death outcomes in relation to UPEC, adjusting for potential confounders using logistic regression analysis; NEC-associated deaths and death free of NEC were not modeled separately because of a lack of adequate case numbers. All factors included in Table 1 were evaluated as potential confounding factors for NEC. Potential confounding factors were examined univariately in relation to NEC; only those factors identified as significant at p < 0.10 were considered in logistic regression models. From this process, six clinical factors were identified as potential confounding factors for NEC: birth weight, GA at birth, singleton versus multiple birth, primiparity versus multiparity, lack of antibiotic use by the mother at the time of delivery, and infant exposure to antibiotics in postnatal life. Given the modest number of NEC cases, we aimed to further reduce the number of confounding factors to be included in models of NEC outcomes. Infant GA and birth weight were highly correlated (rho = 0.7, p < 0.001), and thus, only one of these factors was included in given regression models. Antibiotic use of mother and infant were examined jointly in relation to NEC; the most significant prediction of NEC was found with lack of maternal perinatal antibiotics at delivery combined with high infant antibiotic use (≥7 days in the first two weeks of life), and this variable was used for modeling. Five covariates were entered into the logistic model: high-risk antibiotic use, infant GA (weeks) or infant birth weight (grams), multiparity, multiple birth, and carriage of UPEC. Stepwise removal of factors that were not significant at p <0.10 resulted in a model that included only high-risk antibiotic use (p < 0.001), multiple birth (p = 0.002), and carriage of UPEC (p = 0.001). In order toensure adequate control for immaturity, infant GA was re-entered into the model and kept, as its presence decreased the adjusted OR for UPEC by 13%. In the final logistic model for NEC, the adjusted OR for carriage of UPEC was 6.0 (95% CI 2.0, 18.1; p = 0.002), which remained higher than the OR (Table 4) without adjustment for covariates. The logistic model for NEC or death included the same co-variates and had a somewhat lower OR than for NEC alone (4.6, 95% CI 1.6, 13.0, p = 0.004), but the adjusted OR was again higher than the crude OR. 12 infants developed NEC by the last day of sample availability, day 22 (early), and 15 infants developed NEC after day 22 (late). We analyzed the association between identification of UPEC and odds of NEC that occurred early (versus all controls) and odds of NEC that occurred late (versus all controls). The association between NEC and carriage of UPEC was not significantly influenced by the timing of NEC onset (OR early NEC = 3.7 [1.06, 12.8], p = 0.041; OR late NEC = 4.5 [1.5, 13.9], p = 0.009). Nevertheless, the slightly lower OR for early NEC compared to late NEC is potentially consistent with previous data suggesting that the timing of NEC onset might have somewhat different microbial risk profile (Morrow et al., 2013; Zhou et al., 2015). Thus, the finding that UPEC carriage associated with NEC and death outcomes is highly significant does not appear to be due to confounding factors.

## DISCUSSION

Recent studies have correlated increased relative abundance of Enterobacteriaceae with NEC and suggest this represents a dysbiosis predisposing preterm infants to NEC (Claud et al., 2013; Morrow et al., 2013; Torrazza and Neu, 2013). Epidemic outbreaks have also been documented, suggesting the involvement of infectious agents (Boccia et al., 2001). Culture-based studies have most frequently implicated *E. coli* and *Klebsiella* spp. (Boccia et al., 2001; Hoy et al., 2000) and, to a lesser extent, clostridial species, especially *C. butyricum* (Boccia et al., 2001; Cassir et al., 2015). In our patient population, we identify carriage of uropathogenic subtypes of *E. coli* as a significant contributor to risk of NEC.

### Uropathogenic *E. coli* Is a Risk Factor for NEC and NEC-Associated Death

The major translational findings from this work are that (i) colonization by UPEC is a highly significant risk factor for development of NEC and (ii) UPEC is even more strongly correlated with mortality as an outcome. In our study, 44% of the 27 NEC cases (OR 4.1, p = 0.003) and 52% of the 21 deaths (OR 5.7, p < 0.001) could be attributed to UPEC in comparison to 16% of the 111 controls who were NEC-free survivors. Of the three free-standing NICUs in our study, located in two different cities, each had two or more UPEC-associated cases of NEC. Thus, our findings imply that some proportion of NEC and death cases in other sites may occur as the result of colonization by UPEC strains.

### Early Strain Identification May Reduce NEC Risk

Using assembly-free, metagenomic approaches, we identified *E. coli*-specific accessory gene content that functionally distinguished clades associated with NEC-risk. Furthermore, metage-nomic MLST assignment showed that NEC risk is associated with several important pathogenic MLSTs. Both lines of evidence implicate UPEC. Colonization by UPEC lineages is not restricted to preterm infants; we found that term infants were also colonized by similar MLST as preterm infants (Figure 3). Thus, we suggest that other factors associated with prematurity, such as the hyperinflammatory response of the immature gastrointestinal tract (Nanthakumar et al., 2011), also contribute to the development of NEC in UPEC-colonized infants.

The association of UPEC lineages with NEC-associated death is concerning (OR 10.3, p < 0.001) and underscores the importance of determining the epidemiology of colonization. Colonizing strains may be derived from both maternal and environmental reservoirs such as the neonatal intensive care unit (Adlerberth and Wold, 2009; Brooks et al., 2014; Fryklund et al., 1992). However, both cultivation-based and metagenomic approaches have shown that no particular strain correlates with NEC (Hoy et al., 2000; Raveh-Sadka et al., 2015).

Our finding that greatest NEC risk is associated with a subset of functionally related *E. coli* strains—specifically, uropathogenic *E. coli*—could be overlooked by analysis of specific strain types (e.g., based on MLST or genomic SNP variants) or by considering *E. coli* as a single taxonomic group or OTU (e.g., based on 16S rDNA). Having defined the nature of a risk-associated pathogen, we anticipate our finding will assist epidemiological investigation of the sources of UPEC colonization and NEC risk.

Four preterm twin pairs each harbored the same MLST, suggesting common early colonization. For infants who developed NEC, we observed a correlation of vaginal delivery with NEC-associated death (p = 0.018). In these same infants, the carriage of UPEC in any sample also correlates with vaginal birth (p = 0.057), suggesting that UPEC colonization occurs at birth. If the primary reservoir for infant colonization by UPEC is the maternal gut or urinary tract, an opportunity exists for typing the maternal strain prior to onset of NEC indications. If the strains are primarily nosocomially acquired, then routine surveillance of strains present in the care environment should be emphasized. In either scenario, proactive typing of the colonizing strains would allow assessment of NEC risk and, importantly, risk of NEC-associated death before NEC is indicated.

### *Klebsiella* as a Potential NEC Risk Factor

*E. coli* and *Klebsiella* spp. exhibited similar carriage frequency in this study. However, *E. coli* was unusual in that, when present, it was frequently the dominant organism in the community with a median relative abundance exceeding 80%. In contrast, the next highest median abundance was 48% for *Klebsiella* spp. and only in the days 17–22 collection window. We were not able to assign a species to the *Klebsiella* spp., however, preliminary pan-genomic analysis incorporating *K. pneumoniae* reference genomes indicates the majority of the *Klebsiella* spp. functionally cluster with *K. pneumoniae* subspecies (data not shown). Although *Klebsiella* spp. prevalence did not significantly correlate with NEC, *Klebsiella* spp. subclades may. Torrazza et al. (2013) report that an OTU most similar to *K. pneumoniae* was enriched in the first stool samples collected after birth and correlated with subsequent development of NEC.

### UPEC Antibiotic Resistance May Confound Therapy

Duration of antibiotic treatment has been significantly correlated with increased risk of NEC and death in preterm infants (Alexander et al., 2011; Cotten et al., 2009; Kuppala et al., 2011). Infants with increased antibiotic treatment are likely to have confounding medical conditions, other than specific bacterial colonization, that may also increase their risk of developing NEC. Here, high antibiotic treatment is associated with increased *E. coli* carriage but reduced carriage of *Klebsiella* spp. This suggests that *Klebsiella* may be susceptible to antibiotic treatment and that *E. coli* are more likely to be resistant. A striking finding from our analysis was that NEC infants in whom *Klebsiella* spp. is detected are likely to survive NEC (p = 0.057). Possibly, differential resistance characteristics between the two species contribute to differential outcomes in NEC-associated deaths. Surveys of antibiotic resistance profiles of UPEC isolates report high levels of resistance to ampicillin and gentamicin (Banerjee et al., 2013; Shariff V.A et al., 2013; Wang et al., 2014), a combination frequently used to treat NEC and the combination used in this study. Depending on the colonizing strain, this combination of antibiotics may be less effective for preventing or treating UPEC-associated NEC. Early identification of colonizing subtypes and, ideally, their antibiotic resistance profiles, could inform lifesaving care decisions and the selection of effective antibiotics.

### Dysbiosis and UPEC Establishment

Diversity was lower among infants receiving high antibiotic administration. Diversity was higher at days 17–22 in preterm controls but decreased in infants who developed NEC. Because our measurements are relative abundance, loss of diversity necessarily correlates with increased relative abundance of fewer taxa. Here, we observed increased prevalence of *E. coli* in infant groups where alpha diversity was decreased. High antibiotic administration at days 17–22 was associated with lower relative abundance of Clostridiales and *Veillonella*. NEC was also associated with lower relative abundance of *Veillonella*. Clostridiales and *Veillonella* are relatively enriched in term infants and do not appear to be significantly influenced by mode of birth in preterm infants. Either loss of overall diversity or loss of these key taxa could represent a dysbiosis predisposing infants toward increased abundance of *E. coli* and increased NEC risk.

Alternatively, colonization by *E. coli*, particularly UPEC, may be an event that promotes dysbiosis and increases the likelihood of antibiotic administration. When observed, *E. coli* frequently dominated the community. The relative dominance of *E. coli* may drive the decreased diversity rather than decreased diversity preceding increased *E. coli* abundance. Either way, high antibiotic exposure may favor UPEC strains and reduce the relative abundance and diversity of antibiotic sensitive taxa.

### Alternative Etiologies of NEC

Although we identified UPEC to be the major risk factor for NEC, we also observed NEC cases where *Klebsiella* was dominant. In a recent study, applying both culture-based and qPCR assays, Cassir et al. (2015) found NEC to be significantly associated with the presence of cytotoxic *C. butyricum* strains, reporting that, of 15 NEC cases from 4 different neonatal intensive care units, all carried *C. butyricum*. Metagenomic analysis in our study detected *C. butyricum* in only 1 of 27 preterm infants who developed NEC (3.7%) and in 22 of 117 (18.8%) preterm infants who did not develop NEC. Detection methods differed between the two studies and our study did not utilize an assay specific for *C. butyricum*. Additionally, Cassir et al. (2015) analyzed samples obtained at the time of NEC diagnosis, whereas our sampling was limited to the first weeks of life. Although our study does not implicate *C. butyricum*, it is consistent with the idea that there may be multiple etiologies of NEC.

### Conclusions

The epidemiology, and rapid identification, of *E. coli* strains may be critical to understanding NEC pathology. We demonstrate that metagenomic sequencing in combination with computational approaches for strain-level, functional profiling is an effective, cultivation-free approach for investigating the epidemiology of colonizing *E. coli*. We have shown that detection of UPEC strains indicates risk of NEC and death as an outcome in preterm infants. Future studies should attempt to identify the characteristics of potentially beneficial perinatal antibiotic administration to the mother. Furthermore, proactive typing of the colonizing strains should allow assessment of infants at heightened risk for developing NEC and potentially inform selection of antibiotic therapies to improve outcomes.

## EXPERIMENTAL PROCEDURES

### Subjects

Preterm study infants were enrolled from two level III neonatal intensive care units (NICUs) in Cincinnati, OH, from December 2009 to July 2012, and one level III NICU in Birmingham, AL, from June 2010 to December 2011, as part of a cohort study of novel biomarkers for NEC. The Cincinnati Children’s Hospital Medical Center Institutional Review Board, the University of Alabama Institutional Review Board, the TriHealth Institutional Review Board, and the University of Cincinnati Institutional Review Board approved this study. Written consent was obtained from parents or guardians of study subjects. NEC was defined as Bell’s Stage II or III. Infants with congenital anomalies or spontaneous intestinal perforation were excluded from the study. Data collection used a standardized protocol described previously (Morrow et al., 2013).All NEC cases less than 30 weeks GA were identified, and 4 matched controls were identified per case. NEC incidence by GA of 23–29 weeks was 0.5, 0.21, 0.21, 0.15, 0.21, and 0.11, respectively. NEC incidence and UPEC detection did not significantly differ by birth year, hospital, or NICU (Table S4). Seven NEC cases were enrolled but not included in the study because they either failed to stool prior to NEC or the stool prior to NEC failed to yield sufficient DNA for shotgun sequencing; two of these seven NEC cases died. NEC mortality in this study was 15/27 (55.6%, 95% CI, 36.9%–74.3%). Because NEC-associated mortality typically ranges from 20%–40%, we examined the possibility that mortality was significantly higher than expected. Based on the birth weight distribution of our cases, and applying the birth-weight-specific mortality rates for NEC reported by Fitzgibbons et al. (2009), we calculated the expected mortality of our NEC cases to be 37.0%, which is within the CI of our observed rate. Thus, although the reported mortality is high, it is not evidently higher than expected given the birth weight range of our cases. The median quantity of human milk given over the first 28 days of life was 85% of total enteral feedings. All but three infants received either mother’s own milk, pasteurized human donor milk, or both. Perinatal antibiotic administration is defined as maternal antibiotic use during the admission and prior to delivery of the study infant(s).

### Stool Extraction

Thawed stool was pelleted and resuspended in TE buffer with lysozyme and proteinase K at 0.24 g stool per 100 ul Buffer RLT (QIAGEN) with beta-mercap-toethanol. Samples were homogenized for 3 min in a bead beater with 0.3 g of 0.1 mm glass beads and debris pelleted. DNA was isolated with the QIAGEN AllPrep DNA/RNA Mini Kit (QIAGEN).

### Sequencing Data and Availability

A minimal read length filter of 80 nt was applied to all data used in analysis. Human sequences were removed from the dataset prior to analysis and submission by alignment to the human genome (Genome Reference Consortium Human Build 37 patch release 5; http://www.ncbi.nlm.nih.gov/assembly/GCF_000001405.17/) using the Burrows-Wheeler Aligner (Li and Durbin,2009).

### Sample Selection

Samples were collected at days 3–22 of life. This dataset represents 405 samples from 166 infants, 22 of whom were term (Figure S2). 27 preterm infants developed NEC (median day of NEC onset = 21, range = 7,39). During the study, the initial method of sample extraction was supplanted by a second method that was then applied to the majority of collected samples (Morrow et al., 2013). To avoid potentially confounding influences of extraction protocol, all community-oriented analyses (Table 1; Figures 1, 2, and S3–S6) were performed on infants whose samples were extracted by the second protocol (77% of all infants in study; 105 preterm infants, and all 22 term infants). Analyses of *E.coli* gene content and strain subtypes (Figures 3 and S7; Tables 2–4), and of association of UPEC with NEC and death (Table 4), included all infants in the study.

#### Community Analysis

If multiple samples from the same infant were available with in an analysis window (3–9, 10–16, or 17–22 days), then the sample of latest postnatal collection day was selected. Selection resulted in 262 samples from 127 infants; 16 of the 105 preterm infants developed NEC (median day of NEC onset = 26, range = 10,39).

#### *E.coli* Gene Content and MLST Analysis

We identified 130 samples from 58 infants with reads mapping to *E. coli* genomes (Figure S2); all were subjected to gene content and MLST analysis. When multiple *E. coli* containing samples were available from a single infant, the sample with the highest *E. coli* relative abundance, and >10%, was selected (mean = 79.9%, SD = 26.7) for clade construction and gene-association analysis (Figure 3; Tables 2 and 3). For OR calculation of UPEC as a risk factor for NEC, NEC-associated deaths, and all deaths, all 143 preterm infants were considered (Table 4; Figure S2).

### Community Profiling

Relative taxonomic abundances were determined with MetaPhlAn version 2.0 (Segata et al., 2012; Truong et al., 2015). Resulting species-level abundance estimates were considered for all analysis. Heatmaps were generated with hclust2 (https://bitbucket.org/nsegata/hclust2) using Bray-Curtis dissimilarity for sample clustering. QIIME (Caporaso et al., 2010) package scripts were used to estimate alpha diversity.

### Pangenome-Based Strain-Level Profiling of E.coli from Metagenomes

We generated the *E.coli* pangenome from 116 sequenced reference genomes (Table S3) selected to maximize diversity using PhyloPhlAn (Segata et al., 2013). Genes were clustered into gene families based on 95% sequence similarity using USEARCH (Edgar, 2010), yielding a set of 21,351 gene families establishing the pangenome. Sequences were mapped to all 116 representative *E.coli* genomes with Bowtie2 (version 2.1.0; very-sensitive option). The coverages of all gene locations were extracted with Samtools (Li et al., 2009) and merged as gene-family coverage profiles. A uniform and consistent coverage depth across an expected genome gene set of about 4,600 gene families was used to define the presence of an *E.coli* strain in a sample. Based ona cutoff of half the median depth, coverage levels were converted into gene-family presence and absence profiles that represent the individual gene set of a specific strain captured in a sample. These methods have been implemented as part of the PanPhlAn tool (Scholz et al., 2016) available at http://segatalab.cibio.unitnit/tools/panphlan.

To functionally sub-type *E. coli* strains present in the metagenomes, gene-family presence and absence profiles were hierarchically clustered with Ward’s minimum variance method implemented in the R package, pvclust (http://www.sigmath.es.osaka-u.ac.jp/shimo-lab/prog/pvclust/). Multiscale bootstrap re-sampling was performed with10,000 iterations and the Approximately Unbiased p value (Shimodaira, 2004)is reported. The resulting dendrogram was rendered using Fig Tree software (http://tree.bio.ed.ac.uk/software/figtree/).

### Metagenomic MLST Analysis

We developed a metagenomic approach to exploit the MLST strategy commonly used in cultivation-based typing assays (Maiden et al., 1998). Reads were mapped with Bowtie2 against a database of the known *E. coli* MLST sequences corresponding to distinct alleles of seven genes: *adk*, *fumC*, *gyrB*, *icd*, *mdh*, *purA*, and *recA* (parameters -D 20 -R 3 -N 0 -L 20 -i S,1,0.50). A consensus sequence for each loci was constructed considering the nucleotide with the highest frequency in each position. All samples where all loci obtained a minimum breath of coverage of at least 90% were confidently mapped. For the small fraction of loci with low or non-complete coverage (2.11% of the loci in the positive samples), the best-matching reference allele from the MLST database was used to fill the uncovered positions. Reconstructed consensus alleles were used to determine the most abundant MLST (ST) profile in a sample based on known *E. coli* ST profiles—3,895 known profiles from the University of Warwick Medical School MLST database, http://mlst.warwick.ac.uk/mlst/ (Wirth et al., 2006).

### Statistical Methods

Significance assessment with LEfSe (Segata et al., 2011) first applies the non-parametric factorial Kruskal-Wallis sum-rank test to detect taxa with significant differential abundance with respect to the subject class of interest. LEfSe then uses linear discriminant analysis (LDA) to estimate the effect size of each differentially abundant feature. The alpha value applied for factorial Kruskal-Wallis testing was 0.05; the threshold applied on the logarithmic LDA score for feature discrimination was 2.0. Significance in class comparisons of alpha diversity was calculated with a non-parametric, two-sample t test with 10,000 Monte Carlo simulations implemented in the Qiime 1.8.0 script compare_al-pha_diveristy.py. All other p values reported throughout the manuscript were calculated as two-tailed Fisher’s exact probability test for 2 × 2 contingency tables unless otherwise specified.

## Supplementary Material

1

2

3

4

5

## Figures and Tables

**Table 1:** Characteristics of 144 Preterm and 22 Term Study Infants

Characteristic	NEC Cases	Preterm Control	Term Control
Infants, number	27	117	22
Infant birth weight, median (range), grams*	850 (415,1340)	904 (520,1741)	3476 (2217,4173)
Infant gestational age, median (range), weeks*	26 (23,28)	26 (23, 29)	39 (38, 41)
Male, number (%)	15 (56%)	61 (52%)	11 (50%)
Cesarean section delivery, number (%)	16 (59%)	70 (60%)	10 (45%)
Multiple births, number (%)***	2 (7%)	49 (42%)	2 (9%)
Primiparous, number (%)**	15 (56%)	28 (24%)	7 (32%)
Days infant antibiotic use in first 14 days of life, median (range), days*	7 (0, 14)	3 (0, 14)	0 (0, 0)
Human milk ≥ 75% feedings over the first month of life^a^, number (%)	17 (63%)	86 (74%)	
Maternal antibiotics at delivery (%)*	12 (44%)	79 (68%)	–
Infant with sample available for days 3–9, number (%)	8 (50%)	67 (75%)	22 (100%)
Infant with sample available for days 10–16, number (%)	15 (94%)	81 (91%)	18 (82%)
Infant with sample available for days 17–22, number (%)	7 (44%)	37 (42%)	7 (32%)

* p < 0.05,

** p = 0.002,

*** p = 0.001

a Human milk feeding calculated as the number of days of feedings since birth to the occurrence of NEC, death, or the end of the first month of life, whichever occurred first.

**Table 2:** Selected Genes Associated with Clade 3

KO^a^	p^b^	Gene Description
*Catabolism of 4-hydroxyphenylacetic acid*		
K02508	1.4E-02	hpaA; 4-hydroxyphenylacetate 3-monooxygenase operon regulatory protein
K00483	3.6E-04	hpaB; 4-hydroxyphenylacetate 3-monooxygenase
K00484	3.6E-04	hpaC; flavin reductase (NADH)
K00455	3.6E-04	hpaD; 3,4-dihydroxyphenylacetate 2,3-dioxygenase
K00151	3.6E-04	hpaE; 5-carboxymethyl-2-hydroxymuconic-semialdehyde dehydrogenase
K01826	3.6E-04	hpaF; 5-carboxymethyl-2-hydroxymuconate isomerase
K05921	3.6E-04	hpaG; 4-hydroxyphenylacetate degradation bifunctional isomerase/decarboxylase
K02509	3.6E-04	hpaH; 2-oxo-hept-3-ene-1,7-dioate hydratase
K02510	3.6E-04	hpaI; 4-hydroxy-2-oxoheptanedioate aldolase
K02511	1.4E-02	hpaX; 4-hydroxyphenylacetate permease
*Aerobic catabolism of phenylacetic acid*		
K00146	8.2E-05	feaB; phenylacetaldehyde dehydrogenase
K02609	5.2E-03	paaA; ring-1,2-phenylacetyl-CoA epoxidase subunit
K02610	8.2E-05	paaB; ring-1,2-phenylacetyl-CoA epoxidase subunit
K02611	8.2E-05	paaC; ring-1,2-phenylacetyl-CoA epoxidase subunit
K02612	8.2E-05	paaD; ring-1,2-phenylacetyl-CoA epoxidase subunit
K02613	8.2E-05	paaE; ring-1,2-phenylacetyl-CoA epoxidase subunit
K01692	8.2E-05	paaF; enoyl-CoA hydratase
K15866	8.2E-05	paaG; 2-(1,2-epoxy-1,2-dihydrophenyl) acetyl-CoA isomerase
K02614	8.2E-05	paaI; acyl-CoA thioesterase
K02615	8.2E-05	paaJ; acetyl-CoA acetyltransferase
K01912	8.2E-05	paaK; phenylacetate-CoA ligase
K02616	8.2E-05	paaX; phenylacetic acid degradation operon negative regulatory protein
K02617	2.9E-08	paaY; phenylacetic acid degradation protein
K02618	8.2E-05	paaZ; oxepin-CoA hydrolase / 3-oxo-5, 6-dehydrosuberyl-CoA semialdehyde dehydrogenase
K00276	8.2E-05	tynA; primary-amine oxidase
*Phenylpropanoate/cinnamate degradation*		
K05711	1.5E-06	hcaB; 2,3-dihydroxy-2,3-dihydrophenylpropionate dehydrogenase
K05710	1.5E-06	hcaC; 3-phenylpropionate/trans-cinnamate dioxygenase ferredoxin subunit
K00529	1.5E-06	hcaD; 3-phenylpropionate/trans-cinnamate dioxygenase ferredoxin reductase subunit
K05708	1.5E-06	hcaE; 3-phenylpropionate/trans-cinnamate dioxygenase alpha subunit
K05709	3.3E-05	hcaF; 3-phenylpropionate/trans-cinnamate dioxygenase beta subunit
K05817	1.5E-06	hcaR; hca operon transcriptional activator
K05712	6.0E-04	mhpA; 3-(3-hydroxy-phenyl)propionate hydroxylase
K05713	6.0E-04	mhpB; 2,3-dihydroxyphenylpropionate 1,2-dioxygenase
K05714	6.0E-04	mhpC; 2-hydroxy-6-ketonona-2,4-dienedioic acid hydrolase
K02554	6.0E-04	mhpD; 2-keto-4-pentenoate hydratase
K01666	6.0E-04	mhpE; 4-hydroxy 2-oxovalerate aldolase
K04073	6.0E-04	mhpF; acetaldehyde dehydrogenase
*homology to the hydrogenase hyc operon*		
K12136	1.5E-06	hyfA; hydrogenase-4 component A
K12137	1.5E-06	hyfB; hydrogenase-4 component B
K12138	1.5E-06	hyfC; hydrogenase-4 component C
K12139	1.5E-06	hyfD; hydrogenase-4 component D
K12140	1.5E-06	hyfE; hydrogenase-4 component E
K12141	1.5E-06	hyfF; hydrogenase-4 component F
K12143	3.3E-05	hyfF; hydrogenase-4 component H
K12142	1.5E-06	hyfG; hydrogenase-4 component G
K12144	1.5E-06	hyfI; hydrogenase-4 component I
K12145	1.5E-06	hyfJ; hydrogenase-4 component J
K12146	1.5E-06	hyfR; hydrogenase-4 transcriptional activator

a KEGG orthology (KO) identifier

b p is calculated as two-tailed Fisher’s exact probability. A complete gene list with p values is found in Table S2.

**Table 3:** Selected Genes Associated with NEC Risk Clades 1 and 2

KO^a^	p^b^	Gene Description
*Clade 1 associated*		
K10125	2.1E-12	*dctB*; (C4-dicarboxylate transport) two-component system histidine kinase
K10126	2.1E-12	*dctD*; (C4-dicarboxylate transport) two-component system response regulator
K01451	2.1E-12	*hipO*; hippurate hydrolase
K00016	2.1E-12	L-lactate dehydrogenase
K02765	2.1E-12	PTS system, Fused glucose-specific IIB component
*Clade 1 and 2 associated*		
K08094	7.3E-10	*hxlB*; 6-phospho-3-hexuloisomerase
K03445	7.4E-04	*nepI*; MFS transporter, DHA1 family, purine ribonucleoside efflux pump
*Iron acquisition*		
K16087	8.2E-05	*chuA*: outer membrane heme/hemoglobin/transferrin/lactoferrin receptor protein
K07225	8.2E-05	*chuS*: putative hemin transport protein
K07227	8.2E-05	*chuX* Putative heme iron utilization protein
K06202	1.6E-03	*CyaY* protein
K15721	1.9E-02	*fyuA*; pesticin/yersiniabactin receptor
K04786	5.2E-03	*irp1*; yersiniabactin nonribosomal peptide/polyketide synthase
K04784	1.9E-02	*irp2*; yersiniabactin nonribosomal peptide synthetase
K05374	1.9E-02	*irp4*: ybtT; yersiniabactin synthetase, thioesterase component
K04783	1.9E-02	*irp5*; yersiniabactin salicyl-AMP ligase
K04781	1.9E-02	*irp9*; salicylate synthetase
K07230	3.0E-04	putative iron transporter
K11607	4.5E-04	*sitB*; manganese/iron transport system ATP-binding protein
K11605	4.5E-04	*sitC*; manganese/iron transport system permease protein
K11606	5.1E-05	*sitD*; manganese/iron transport system permease protein
K05372	5.2E-03	*ybtA*; AraC family transcriptional regulator
*Phosphotransferase systems*		
K02538	1.4E-02	*manR*; activator of the mannose operon, transcriptional antiterminator
K02768	1.4E-02	PTS system, fructose-specific IIA component
K02769	1.4E-02	PTS system, fructose-specific IIB component
K02765	1.5E-06	PTS system, Fused glucose-specific IIB component
K02812	8.2E-05	*sorF*; PTS system, sorbose-specific IIA component
K02813	7.1E-04	*sorB*; PTS system, sorbose-specific IIB component
K02814	7.1E-04	*sorA*; PTS system, sorbose-specific IIC component
K02815	7.1E-04	*sorD*; PTS system, sorbose-specific IID component
K11189	7.1E-04	*fryA*; PTS-HPR; phosphocarrier protein
*Serine*		
K01753	1.6E-03	*dsdA*; D-serine dehydratase
K13636	1.8E-02	*dsdC*; LysR family transcriptional regulator, D-serine deaminase activator
K13629	3.5E-02	*dsdX*; D-serine transporter

a KEGG Orthology (KO) identifier

b p is calculated as two-tailed Fisher’s exact probability. A complete gene list with p values is found in Table S2.

**Table 4:** Carriage of UPEC and Odds of NEC and Death Outcomes

Outcomes	No. UPEC/Cases (%)	Unadjusted OR (95% CI), p value	Adjusted OR (95% CI), p value
NEC	12/27 (44.4)	4.1 (1.5, 11.2) *0.003*	6.0 (2.0, 18.1) *0.002*
NEC-associated deaths	10/15 (66.7)	10.3 (2.8, 42.2) <*0.001*	–
All deaths	11/21 (52.4)	5.7 (1.8, 17.2) <*0.001*	–
NEC or death	13/33 (39.4)	3.4 (1.3, 8.6) *0.007*	4.6 (1.6, 13.0) *0.004*
Controls^a^	18/111 (16.2)	1.0	1.0

Adjusted OR calculated from logistic regression models including UPEC carriage, multiple birth, maternal and infant antibiotic use, and infant gestational age.

a Controls were defined as preterm infants who survived free of NEC.

## Highlights

- Uropathogenic *E. coli* is a risk factor for necrotizing enterocolitis and death
- Assembly-free metagenomic methods enable strain-level analysis and epidemiology
- Identification of *E. coli* strains may be critical to understanding NEC pathology
