# Supplementary material for: Metagenomic Sequencing with Strain-Level Resolution Implicates Uropathogenic E. coli in Necrotizing Enterocolitis and Mortality in Preterm Infants
Source: Cell Rep. Author manuscript; Available in PMC 2016 Apr 4. (PMC4819403; doi:10.1016/j.celrep.2016.03.015)
Supplement: 1 [file NIHMS770918-supplement-1.pdf]

Cell Reports, Volume *14*

Supplemental Information

Metagenomic Sequencing with Strain-Level Resolution Implicates Uropathogenic *E. coli* in Necrotizing Enterocolitis and Mortality in Preterm Infants

Doyle V. Ward, Matthias Scholz, Moreno Zolfo, Diana H. Taft, Kurt R. Schibler, Adrian Tett, Nicola Segata, and Ardythe L. Morrow

Figure S1

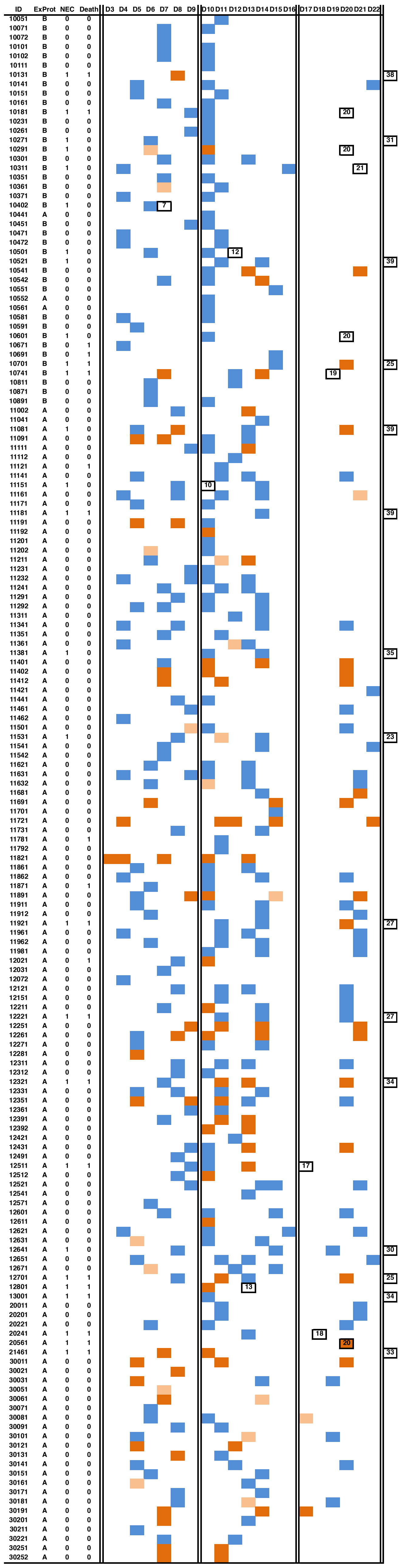
ID
ExProt
NEC
Death
D3 D4 D5 D6 D7 D8 D9
D10 D11 D12 D13 D14 D15 D16
D17 D18 D19 D20 D21 D22

Figure S2

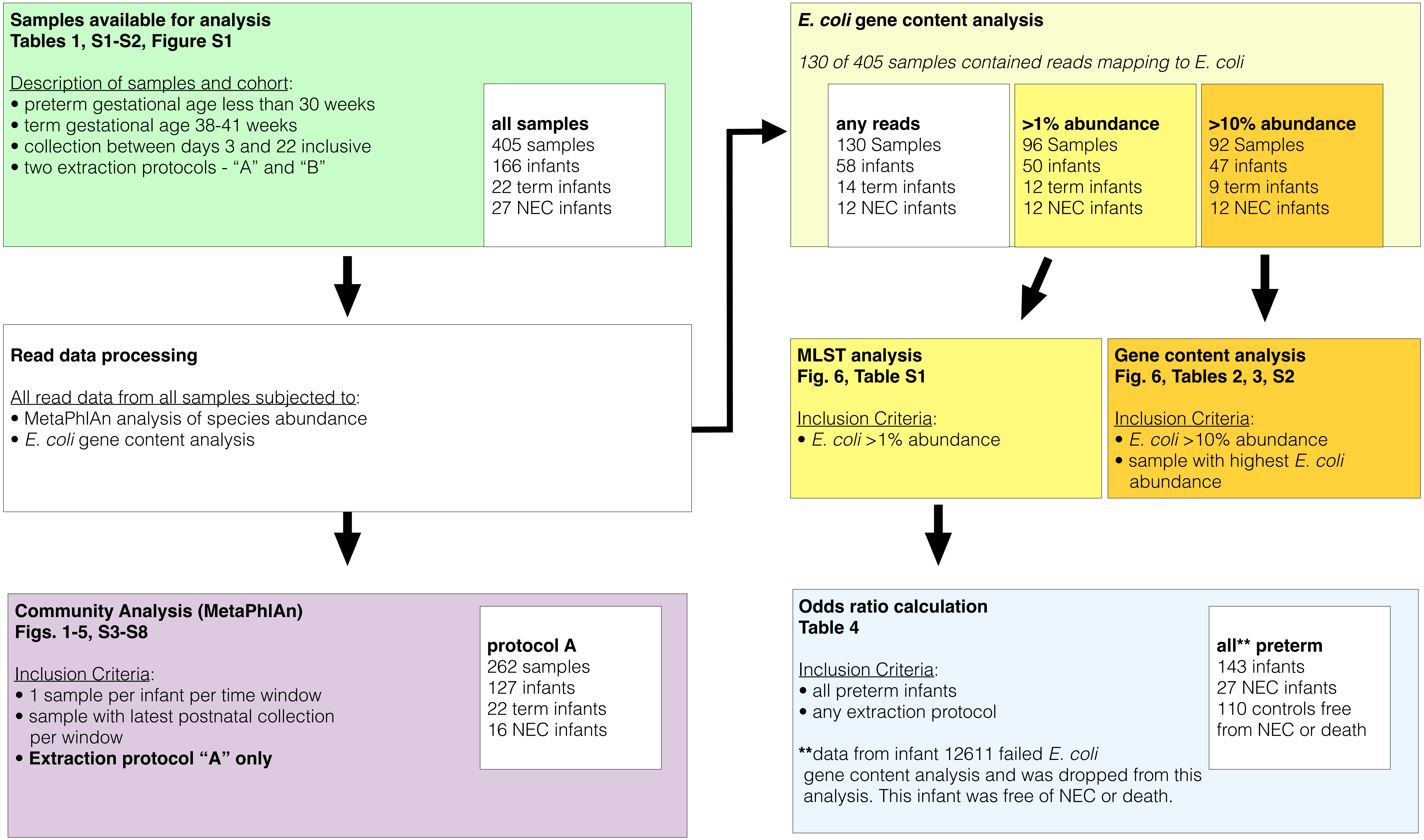
Samples available for analysis
Tables 1, S1-S2, Figure S1
Description of samples and cohort:
• preterm gestational age less than 30 weeks
• term gestational age 38-41 weeks
• collection between days 3 and 22 inclusive
• two extraction protocols - "A" and "B"
all samples
405 samples
166 infants
22 term infants
27 NEC infants
Read data processing
All read data from all samples subjected to:
• MetaPhlAn analysis of species abundance
• E. coli gene content analysis
Community Analysis (MetaPhlAn)
Figs. 1-5, S3-S8
Inclusion Criteria:
• 1 sample per infant per time window
• sample with latest postnatal collection per window
• Extraction protocol "A" only
protocol A
262 samples
127 infants
22 term infants
16 NEC infants
E. coli gene content analysis
130 of 405 samples contained reads mapping to E. coli
any reads
130 Samples
58 infants
14 term infants
12 NEC infants
>1% abundance
96 Samples
50 infants
12 term infants
12 NEC infants
>10% abundance
92 Samples
47 infants
9 term infants
12 NEC infants
MLST analysis
Fig. 6, Table S1
Inclusion Criteria:
• E. coli >1% abundance
Gene content analysis
Fig. 6, Tables 2, 3, S2
Inclusion Criteria:
• E. coli >10% abundance
• sample with highest E. coli abundance
Odds ratio calculation
Table 4
Inclusion Criteria:
• all preterm infants
• any extraction protocol
**data from infant 12611 failed E. coli gene content analysis and was dropped from this analysis. This infant was free of NEC or death.
all** preterm
143 infants
27 NEC infants
110 controls free from NEC or death

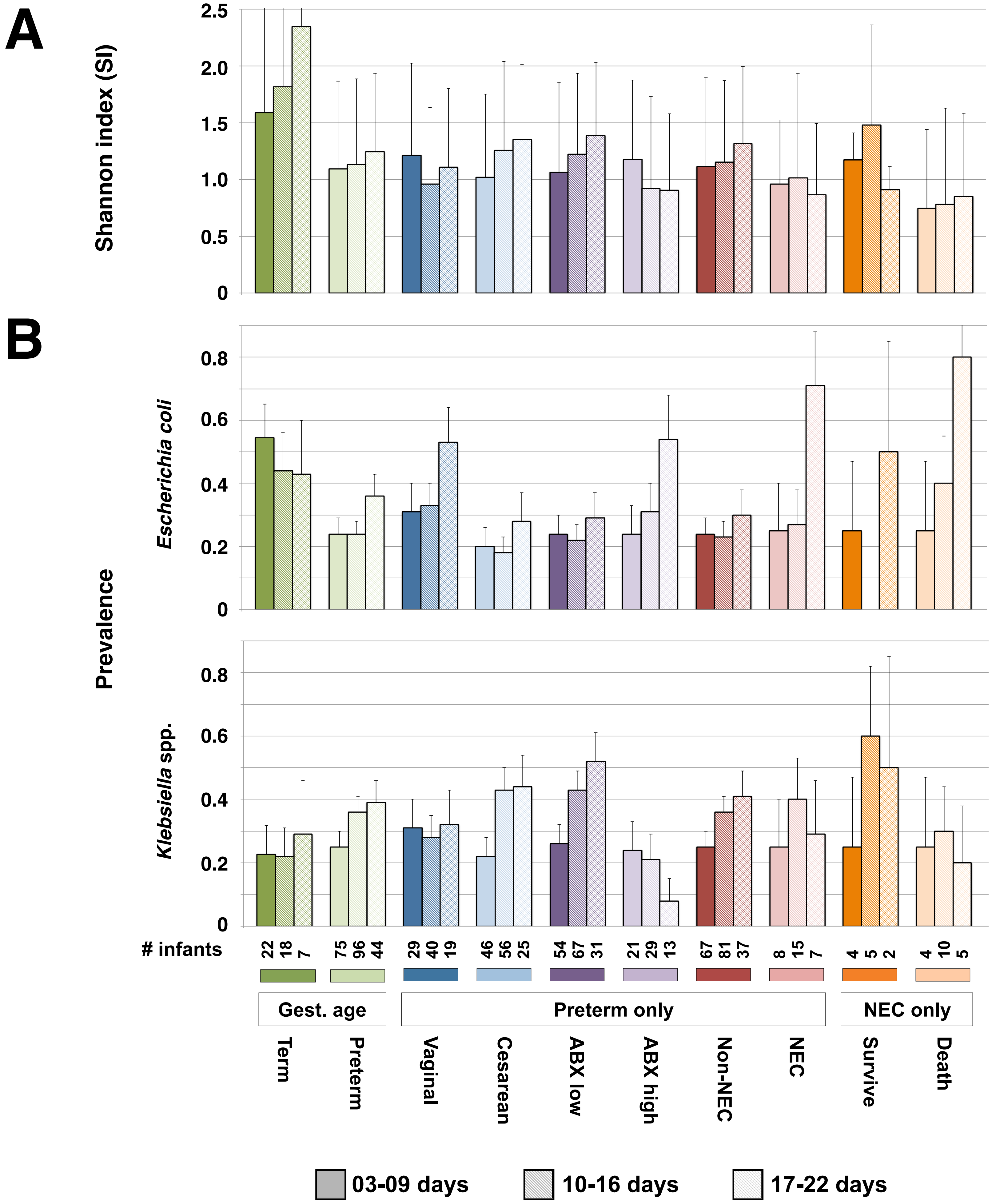
A
B
Shannon index (SI)
2.5
2.0
1.5
1.0
0.5
0
Prevalence
Escherichia coli
Klebsiella spp.
0.8
0.6
0.4
0.2
0
infants
22 18 7
75 96 44
29 40 19
46 56 25
54 67 31
21 29 13
67 81 37
8 15 7
4 5 2
4 10 5
Gest. age
Preterm only
NEC only
Term
Preterm
Vaginal
Cesarean
ABX low
ABX high
Non-NEC
NEC
Survive
Death
03-09 days
10-16 days
17-22 days

Figure S4

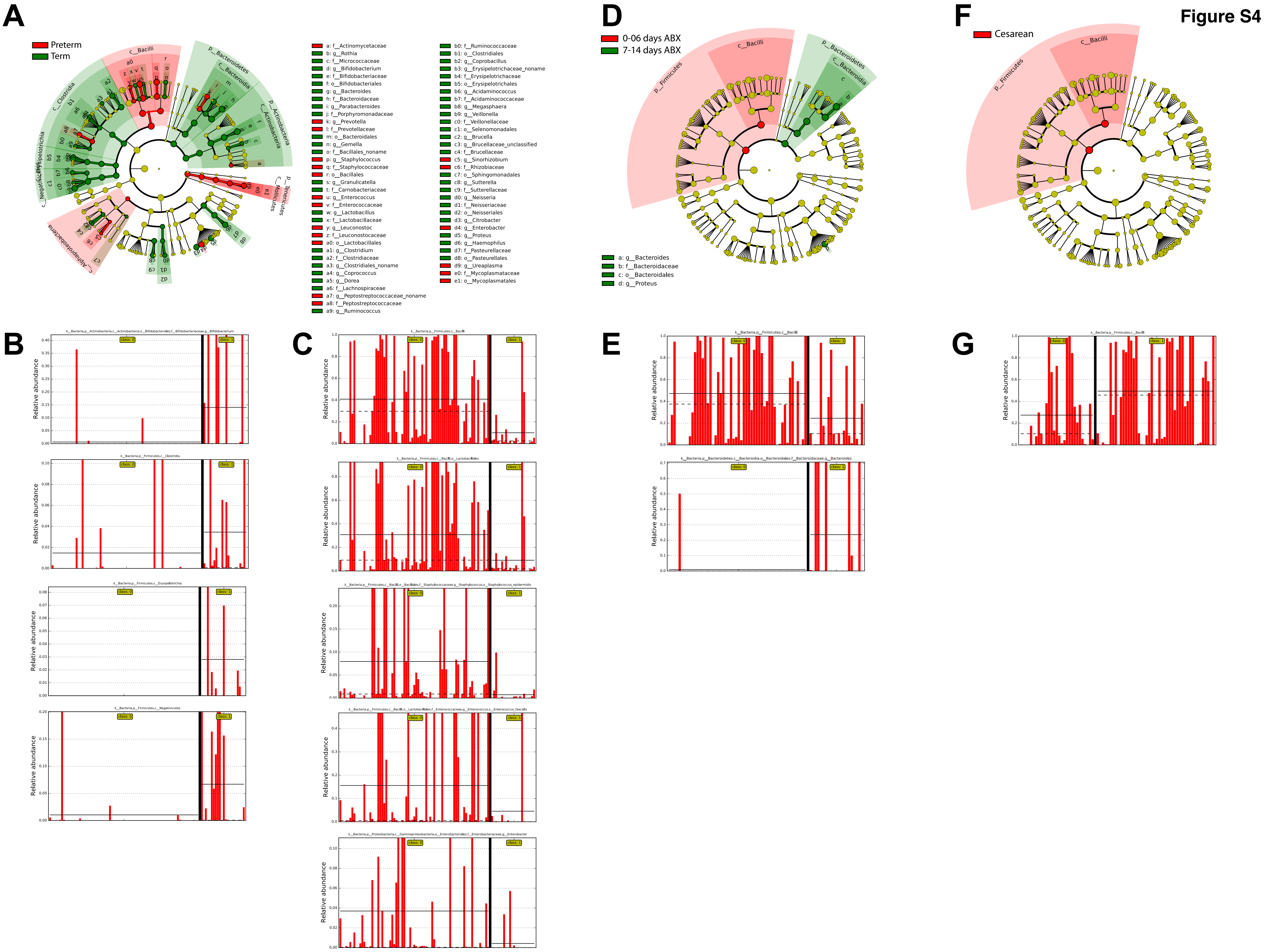

A
Preterm
Term
a: f__Actinomycetaceae
b: g__Rothia
c: f__Micrococcaceae
d: g__Bifidobacterium
e: f__Bifidobacteriaceae
f: o__Bifidobacteriales
g: g__Bacteroides
h: f__Bacteroidaceae
i: g__Parabacteroides
j: f__Porphyromonadaceae
k: g__Prevotella
l: f__Prevotellaceae
m: o__Bacteroidales
n: g__Gemella
o: f__Bacillales_noname
p: g__Staphylococcus
q: f__Staphylococcaceae
r: o__Bacillales
s: g__Granulicatella
t: f__Carnobacteriaceae
u: g__Enterococcus
v: f__Enterococcaceae
w: g__Lactobacillus
x: f__Lactobacillaceae
y: g__Leuconostoc
z: f__Leuconostocaceae
a0: o__Lactobacillales
a1: g__Clostridium
a2: f__Clostridiaceae
a3: f__Clostridiales_noname
a4: g__Coprococcus
a5: g__Dorea
a6: f__Lachnospiraceae
a7: g__Peptostreptococcaceae_noname
a8: f__Peptostreptococcaceae
a9: g__Ruminococcus
b0: f__Ruminococcaceae
b1: o__Clostridiales
b2: g__Coprobacillus
b3: g__Erysipelotrichaceae_noname
b4: f__Erysipelotrichaceae
b5: o__Erysipelotrichales
b6: g__Acidaminococcus
b7: f__Acidaminococcaceae
b8: g__Megasphaera
b9: g__Veillonella
c0: f__Veillonellaceae
c1: o__Selenomonadales
c2: g__Brucella
c3: g__Brucellaceae_unclassified
c4: f__Brucellaceae
c5: g__Sinorhizobium
c6: f__Rhizobiaceae
c7: o__Sphingomonadales
c8: g__Sutterella
c9: f__Sutterellaceae
d0: g__Neisseria
d1: f__Neisseriaceae
d2: o__Neisseriales
d3: g__Citrobacter
d4: g__Enterobacter
d5: g__Proteus
d6: g__Haemophilus
d7: f__Pasteurellaceae
d8: o__Pasteurellales
d9: g__Ureaplasma
e0: f__Mycoplasmataceae
e1: o__Mycoplasmatales
D
0-06 days ABX
7-14 days ABX
a: g__Bacteroides
b: f__Bacteroidaceae
c: o__Bacteroidales
d: g__Proteus
F
Cesarean
B
C
E
G
Relative abundance

Figure S5

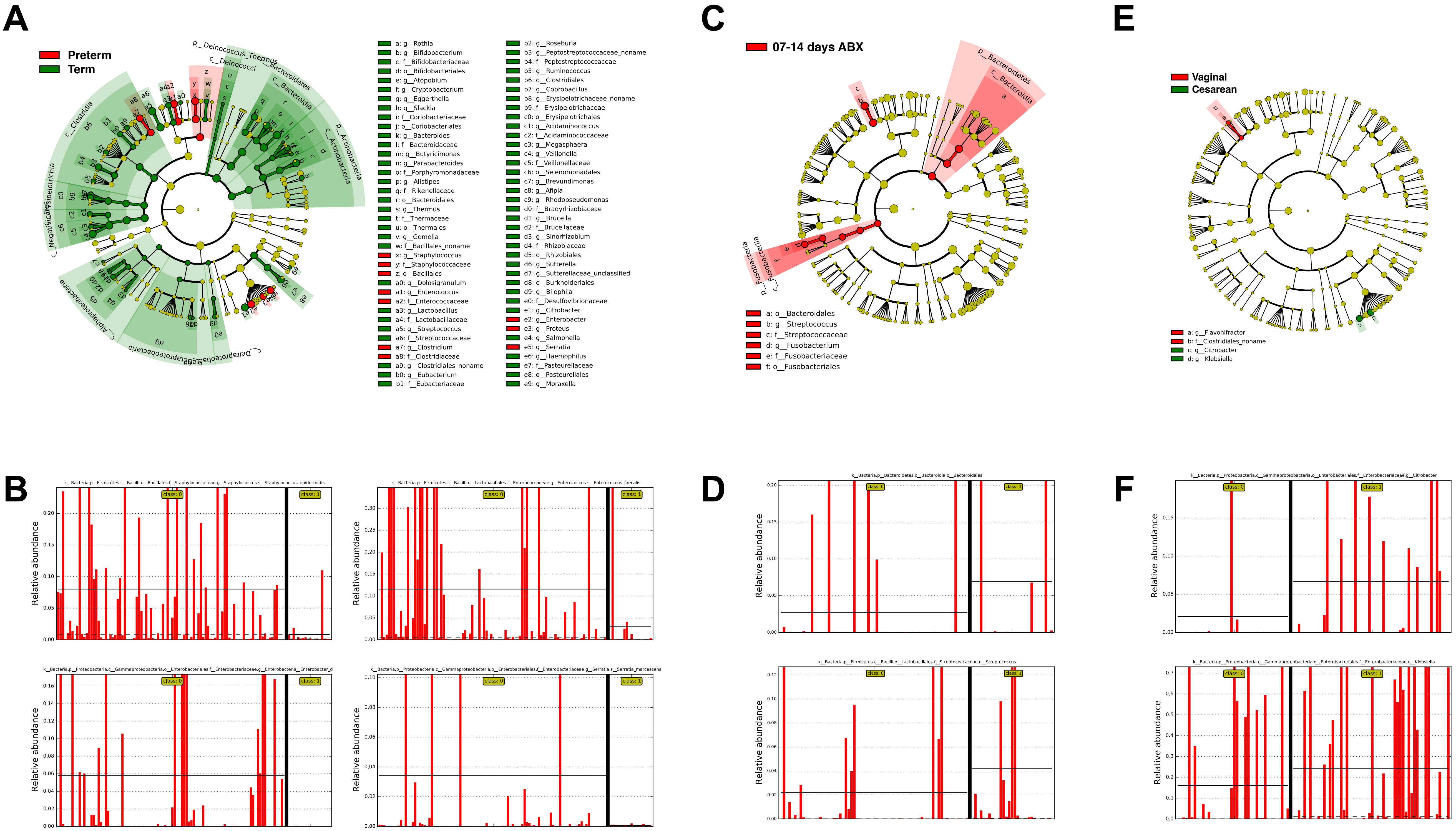
A
Preterm
Term
a: g__Rothia
b: g__Bifidobacterium
c: f__Bifidobacteriaceae
d: o__Bifidobacteriales
e: g__Atopobium
f: g__Cryptobacterium
g: g__Eggerthella
h: g__Slackia
i: f__Coriobacteriaceae
j: o__Coriobacteriales
k: g__Bacteroides
l: f__Bacteroidaceae
m: g__Butyricimonas
n: g__Parabacteroides
o: f__Porphyromonadaceae
p: g__Alistipes
q: f__Rikenellaceae
r: o__Bacteroidales
s: g__Thermus
t: f__Thermaceae
u: o__Thermales
v: g__Gemella
w: f__Bacillales_noname
x: g__Staphylococcus
y: f__Staphylococcaceae
z: o__Bacillales
a0: g__Dolosigranulum
a1: g__Enterococcus
a2: f__Enterococcaceae
a3: g__Lactobacillus
a4: f__Lactobacillaceae
a5: g__Streptococcus
a6: f__Streptococcaceae
a7: g__Clostridium
a8: f__Clostridiaceae
a9: g__Clostridiales_noname
b0: g__Eubacterium
b1: f__Eubacteriaceae
b2: g__Roseburia
b3: g__Peptostreptococcaceae_noname
b4: f__Peptostreptococcaceae
b5: g__Ruminococcus
b6: o__Clostridiales
b7: g__Coprobacillus
b8: g__Erysipelotrichaceae_noname
b9: f__Erysipelotrichaceae
c0: o__Erysipelotrichales
c1: g__Acidaminococcus
c2: f__Acidaminococcaceae
c3: g__Megasphaera
c4: g__Veillonella
c5: f__Veillonellaceae
c6: o__Selenomonadales
c7: g__Brevundimonas
c8: g__Afipia
c9: g__Rhodopseudomonas
d0: f__Bradyrhizobiaceae
d1: g__Brucella
d2: f__Brucellaceae
d3: g__Sinorhizobium
d4: f__Rhizobiaceae
d5: o__Rhizobiales
d6: g__Sutterella
d7: g__Sutterellaceae_unclassified
d8: o__Burkholderiales
d9: g__Bilophila
e0: f__Desulfovibrionaceae
e1: g__Citrobacter
e2: g__Enterobacter
e3: g__Proteus
e4: g__Salmonella
e5: g__Serratia
e6: g__Haemophilus
e7: f__Pasteurellaceae
e8: o__Pasteurellales
e9: g__Moraxella
B
k__Bacteria.p__Firmicutes.c__Bacilli.o__Bacillales.f__Staphylococcaceae.g__Staphylococcus.s__Staphylococcus_epidermidis
k__Bacteria.p__Firmicutes.c__Bacilli.o__Lactobacillales.f__Enterococcaceae.g__Enterococcus.s__Enterococcus_faecalis
k__Bacteria.p__Proteobacteria.c__Gammaproteobacteria.o__Enterobacteriales.f__Enterobacteriaceae.g__Enterobacter.s__Enterobacter_cl
k__Bacteria.p__Proteobacteria.c__Gammaproteobacteria.o__Enterobacteriales.f__Enterobacteriaceae.g__Serratia.s__Serratia_marcescens
Relative abundance
class: 0
class: 1
C
07-14 days ABX
a: o__Bacteroidales
b: g__Streptococcus
c: f__Streptococcaceae
d: g__Fusobacterium
e: f__Fusobacteriaceae
f: o__Fusobacteriales
D
k__Bacteria.p__Bacteroidetes.c__Bacteroidia.o__Bacteroidales
k__Bacteria.p__Firmicutes.c__Bacilli.o__Lactobacillales.f__Streptococcaceae.g__Streptococcus
E
Vaginal
Cesarean
a: g__Flavonifractor
b: f__Clostridiales_noname
c: g__Citrobacter
d: g__Klebsiella
F
k__Bacteria.p__Proteobacteria.c__Gammaproteobacteria.o__Enterobacteriales.f__Enterobacteriaceae.g__Citrobacter
k__Bacteria.p__Proteobacteria.c__Gammaproteobacteria.o__Enterobacteriales.f__Enterobacteriaceae.g__Klebsiella

Figure S6

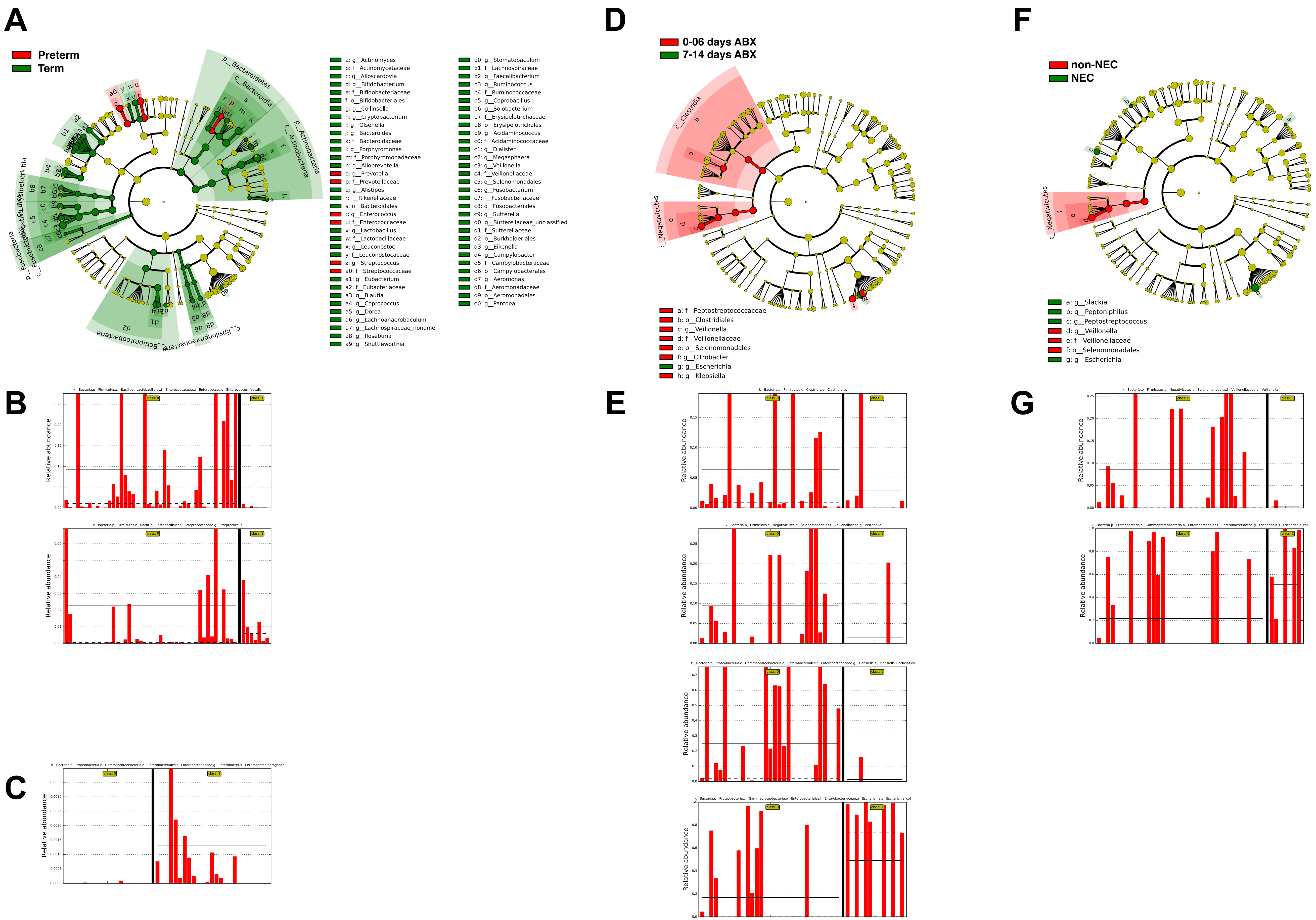
A
Preterm
Term
a: g__Actinomyces
b: f__Actinomycetaceae
c: g__Alloscardovia
d: g__Bifidobacterium
e: f__Bifidobacteriaceae
f: o__Bifidobacteriales
g: g__Collinsella
h: g__Cryptobacterium
i: g__Olsenella
j: g__Bacteroides
k: f__Bacteroidaceae
l: g__Porphyromonas
m: f__Porphyromonadaceae
n: g__Alloprevotella
o: g__Prevotella
p: f__Prevotellaceae
q: g__Alistipes
r: f__Rikenellaceae
s: o__Bacteroidales
t: g__Enterococcus
u: f__Enterococcaceae
v: g__Lactobacillus
w: f__Lactobacillaceae
x: g__Leuconostoc
y: f__Leuconostocaceae
z: g__Streptococcus
a0: f__Streptococcaceae
a1: g__Eubacterium
a2: f__Eubacteriaceae
a3: g__Blautia
a4: g__Coprococcus
a5: g__Dorea
a6: g__Lachnoanaerobaculum
a7: g__Lachnospiraceae_noname
a8: g__Roseburia
a9: g__Shuttleworthia
b0: g__Stomatobaculum
b1: f__Lachnospiraceae
b2: g__Faecalibacterium
b3: g__Ruminococcus
b4: f__Ruminococcaceae
b5: g__Coprobacillus
b6: g__Solobacterium
b7: f__Erysipelotrichaceae
b8: o__Erysipelotrichales
b9: g__Acidaminococcus
c0: f__Acidaminococcaceae
c1: g__Dialister
c2: g__Megasphaera
c3: g__Veillonella
c4: f__Veillonellaceae
c5: o__Selenomonadales
c6: g__Fusobacterium
c7: f__Fusobacteriaceae
c8: o__Fusobacteriales
c9: g__Sutterella
d0: g__Sutterellaceae_unclassified
d1: f__Sutterellaceae
d2: o__Burkholderiales
d3: g__Eikenella
d4: g__Campylobacter
d5: f__Campylobacteraceae
d6: o__Campylobacterales
d7: g__Aeromonas
d8: f__Aeromonadaceae
d9: o__Aeromonadales
e0: g__Pantoea
B
Relative abundance
C
Relative abundance
D
0-06 days ABX
7-14 days ABX
a: f__Peptostreptococcaceae
b: o__Clostridiales
c: g__Veillonella
d: f__Veillonellaceae
e: o__Selenomonadales
f: g__Citrobacter
g: g__Escherichia
h: g__Klebsiella
E
Relative abundance
F
non-NEC
NEC
a: g__Slackia
b: g__Peptoniphilus
c: g__Peptostreptococcus
d: g__Veillonella
e: f__Veillonellaceae
f: o__Selenomonadales
g: g__Escherichia
G
Relative abundance

Figure S7

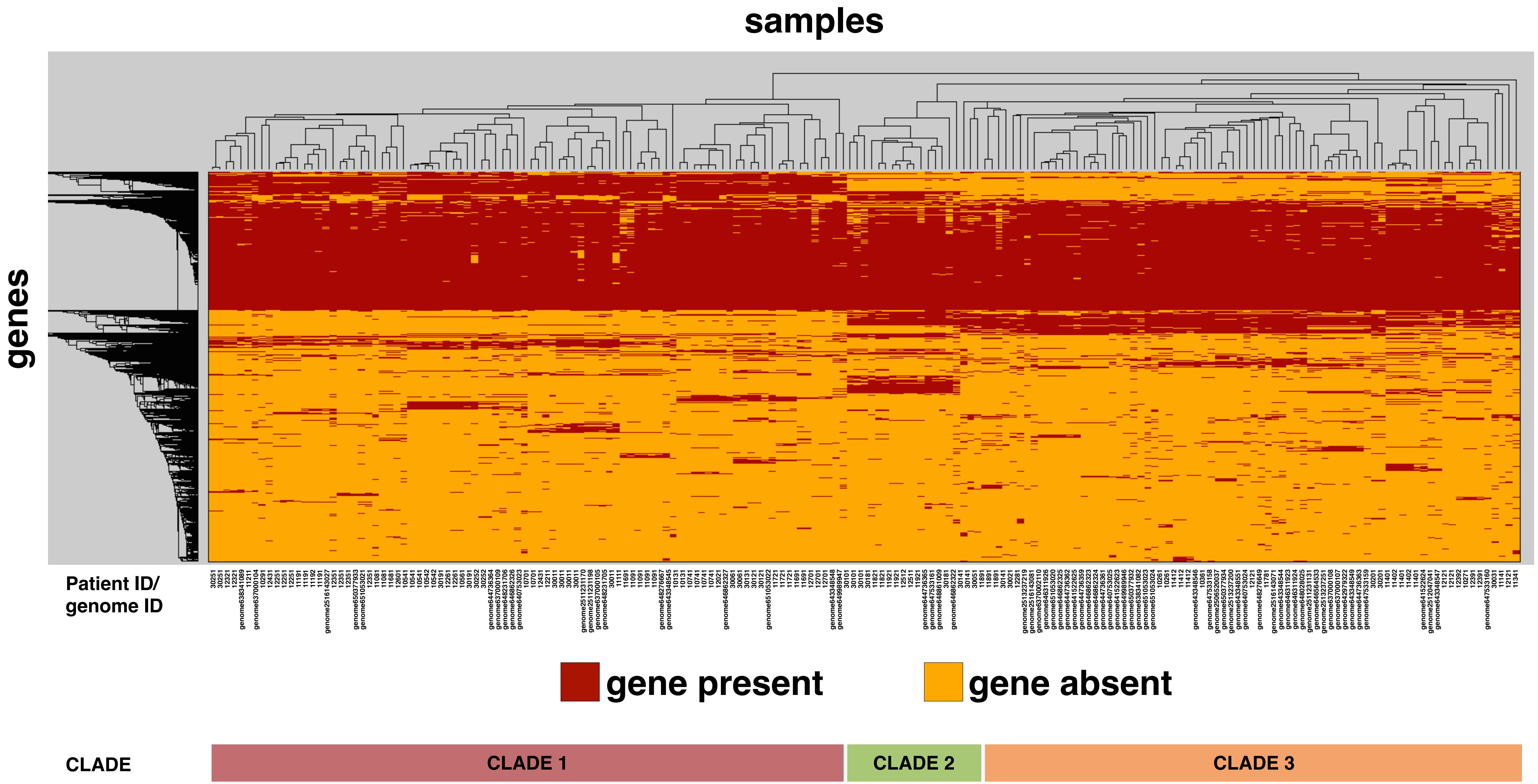
samples
genes
Patient ID/
genome ID
gene present
gene absent
CLADE
CLADE 1
CLADE 2
CLADE 3

SUPPLEMENTAL FIGURE LEGENDS

Figure S1. Samples available for analysis related to Experimental Procedures. Samples available per infant are plotted by day postpartum and color-coded to indicate relative abundance of *E. coli* at <1% (blue), 1-<10% (light orange), and at least 10% (orange). Samples are divided by analysis group (days 3-9, days 10-16 and days 17-22). Day of NEC onset is indicated by the number of the day postpartum in an outlined box. Also indicated is which of the two extraction protocols applied, as well as whether the infant developed NEC or died. Patient IDs for term infants begin with “3”.

Figure S2. Samples flow chart for analysis related to Experimental Procedures. General characteristics of all 405 samples available for analysis are described (green). Read data from all samples was analyzed by MetaPhlAn analysis and also mapped to *E. coli* genomes for gene content analysis (white). All samples extracted under protocol A were included in community analysis (Purple). All samples with data mapping to *E. coli* genomes were considered for *E. coli* gene content analysis (light yellow); several samples did not contain enough read data to determine MLST type or call gene content. Samples with at least 1% relative abundance of *E. coli* and had a determined MLST type were considered for MLST analysis (yellow). Samples with at least 10% relative abundance of *E. coli* and represented the sample with highest *E. coli* abundance for any sample from an individual infant were included in gene content analysis (orange). Odds ratio calculations with respect to UPEC included all preterm infants (blue) and used criteria of MLST analysis (yellow) to determine UPEC presence in an infant.

Figure S3. Clinical factors associated with community diversity and prevalence of *E. coli* and *Klebsiella* spp related to Figure 1. (A) Shannon's index of alpha diversity per infant group. Standard deviation is indicated by error bar. (B) Prevalence of *E. coli* and *Klebsiella* spp. per infant group. Prevalence is defined as the frequency each organism is carried in infants at greater than 1% relative abundance. The number of infants per group is indicated at bottom of figure. Date for each of the three collection windows is presented from left to right as day 3-9 (dark), day 10-16 (medium), and day 17-22 (light). Except for the 'Term' group (darker green), all other groups include only preterm infants less than 30 weeks gestational age.

Figure S4. Taxonomic biomarkers enriched in infants sampled at days 3-9 postpartum related to Figure 1. (A) Cladogram generated using LEfSe (Segata et al., 2011) indicates taxonomies found to be significantly enriched in either preterm or term infants. (B) Taxonomies significantly enriched in Term infants ("class:1", right side). (C) Taxonomies significantly enriched in Preterm infants ("class:0", left side). (D) Cladogram indicates taxonomies found to be significantly enriched in preterm infants with low (0-6 days) or high (7-14 days) antibiotic administration during their first 14 days of life. (E) Taxonomies significantly enriched in preterm infants with high antibiotic administration ("class:1", right side) or low antibiotic administration ("class:0", left side). (F) Cladogram indicating taxonomies enriched in preterm Cesarean births. (G) Bacilla were significantly enriched in preterm Cesarean births ("class:1", right side). Histograms plot relative abundance for each patient and were generated using LEfSe. Relative abundance scales differ for each plot. Median (dashed line) and Mean (solid line) relative abundances per group are indicated.

Figure S5. Taxonomic biomarkers enriched in infants sampled at days 10-16 postpartum related to Figure 1. (A) Cladogram generated using LEfSe (Segata et al., 2011) indicates taxonomies found to be significantly enriched in either preterm or term infants. (B) Taxonomies significantly enriched in preterm infants ("class:0", left side). (C) indicates taxonomies found to be significantly enriched in preterm infants with high (7-14 days) antibiotic administration. (D) Taxonomies significantly enriched in preterm infants receiving 7-14 days ("class:1", right side) and 0-6 days ("class:0", left side) antibiotic administration. (E) Cladogram indicating taxonomies enriched in preterm vaginal or Cesarean births. (F) Taxonomies significantly enriched in preterm Cesarean births ("class:1", right side). Histograms plot relative abundance for each patient and were generated using LEfSe. Relative abundance scales differ for each plot. Median (dashed line) and Mean (solid line) relative abundances per group are indicated.

Figure S6. Taxonomic biomarkers enriched in infants sampled at days 17-22 postpartum related to Figure 1. (A) Cladogram generated using LEfSe (Segata et al., 2011) indicates taxonomies found to be significantly enriched in either preterm or term infants. (B) Taxonomies significantly enriched in preterm infants ("class:0", left side). (C) *Enterobacter aerogenes* is enriched in preterm infants delivered by Cesarean ("class:1; right side). (D) Cladogram indicates taxonomies found to be significantly enriched in preterm infants with low (0-6 days) or high (7-14 days) antibiotic administration. (E) Taxonomies significantly enriched in preterm infants with 0-6 days ("class:0", left side) and 7-14 days ("class:1", right side) antibiotic administration. (F) Cladogram indicating taxonomies enriched in preterm infants who did or did not develop NEC. (G) *E. coli* is significantly enriched in preterm NEC infants ("class:1", right side) while *Veillonella* is enriched in preterm infants who did not develop NEC ("class:0"; left side). Histograms plot relative abundance for each patient and were generated using LEfSe. Relative abundance scales differ for each plot. Median (dashed line) and Mean (solid line) relative abundances per group are indicated.

Figure S7. Infant samples clustered by *E. coli* gene content indicate samples from same infant collected over time contain the same strain of *E. coli* related to Figure 3. Infant samples subjected to *E. coli* gene content analysis were clustered using gene present/absence data using the Bray-Curtis dissimilarity metric. Reference *E. coli* genomes were also included in the cluster analysis. Patient and genome IDs are indicated. Clade identities relating to Figure 3 are also indicated.
